# A Large Gene Network in Immature Erythroid Cells Is Controlled by the Myeloid and B Cell Transcriptional Regulator PU.1

**DOI:** 10.1371/journal.pgen.1001392

**Published:** 2011-06-09

**Authors:** Sandeep N. Wontakal, Xingyi Guo, Britta Will, Minyi Shi, Debasish Raha, Milind C. Mahajan, Sherman Weissman, Michael Snyder, Ulrich Steidl, Deyou Zheng, Arthur I. Skoultchi

**Affiliations:** 1 Department of Cell Biology, Albert Einstein College of Medicine, Bronx, New York, United States of America; 2 The Saul R. Korey Department of Neurology, Albert Einstein College of Medicine, Bronx, New York, United States of America; 3 Department of Molecular, Cellular, and Developmental Biology, Yale University, New Haven, Connecticut, United States of America; 4 Department of Genetics, Stanford University, Stanford, California, United States of America; 5 Department of Genetics, Yale University School of Medicine, New Haven, Connecticut, United States of America; 6 Departments of Genetics and Neuroscience, Albert Einstein College of Medicine, Bronx, New York, United States of America; Children's Hosptial of Philadelphia, United States of America

## Abstract

PU.1 is a hematopoietic transcription factor that is required for the development of myeloid and B cells. PU.1 is also expressed in erythroid progenitors, where it blocks erythroid differentiation by binding to and inhibiting the main erythroid promoting factor, GATA-1. However, other mechanisms by which PU.1 affects the fate of erythroid progenitors have not been thoroughly explored. Here, we used ChIP-Seq analysis for PU.1 and gene expression profiling in erythroid cells to show that PU.1 regulates an extensive network of genes that constitute major pathways for controlling growth and survival of immature erythroid cells. By analyzing fetal liver erythroid progenitors from mice with low PU.1 expression, we also show that the earliest erythroid committed cells are dramatically reduced *in vivo*. Furthermore, we find that PU.1 also regulates many of the same genes and pathways in other blood cells, leading us to propose that PU.1 is a multifaceted factor with overlapping, as well as distinct, functions in several hematopoietic lineages.

## Author Summary

Cellular identities are established by master regulatory transcription factors that promote cell type–specific gene expression. In some instances such factors also inhibit differentiation of alternative, closely related lineages. PU.1 is an Ets family transcription factor that is required for myeloid and B cell development. PU.1 is also expressed in red blood cell progenitors where it blocks erythroid terminal differentiation. One mechanism used by PU.1 to block red blood cell differentiation is by binding to and inhibiting the erythroid master regulator GATA-1. Here, we describe another mechanism utilized by PU.1 in erythroid progenitors. Using chromatin immunoprecipitation and high-throughput sequencing (ChIP-Seq) combined with gene expression profiling of erythroid progenitors, we show that PU.1 controls a large gene network in immature erythroid cells, including genes in pathways involved in regulating growth, survival, and differentiation of these cells. We also find that PU.1 controls many of the same genes and pathways in other blood cells. The results indicate that, in addition to activating lineage-specific genes, master regulatory transcription factors, like PU.1, also control numerous, widely expressed genes in multiple cell lineages.

## Introduction

Cellular identities are established through the actions of master regulatory transcription factors. In addition to promoting their lineage-specific gene expression programs, these factors may also inhibit the transcriptional programs of alternative cell lineages [1]. These inhibitory functions may serve to ensure that genes of closely related lineages are not mis-expressed [2], [3]. The mechanisms used by such transcription factors to inhibit alternative lineage-specific gene expression are not well understood. The mutual antagonism between hematopoietic master regulators PU.1 and GATA-1 has served as an important paradigm, both for understanding lineage specification as well as these types of inhibitory interactions.

PU.1 is an Ets family transcription factor that is required for the development of myeloid and B-cells [4], [5]. GATA-1 is a Zn-finger DNA binding protein that is required for the development of erythrocytes and megakaryocytes [6]. These two factors have a particularly close developmental relationship because they direct lineage commitment from common multipotential progenitors. Indeed, PU.1 and GATA-1 physically interact and repress each other's transcriptional activation and lineage specification functions [7]–[9].

Although PU.1 is highly expressed in myeloid and B-cells, it is also normally present in immature erythroid cells [10]–[12]. Down regulation of PU.1 is required for erythroid terminal differentiation [11]–[13]. This property has been conserved throughout vertebrate evolution [7], [14], [15]. The Sfpi1 locus encoding PU.1 is also a frequent target for integration by the spleen-focus-forming virus during Friend leukemia virus-induced murine erythroleukemia (MEL) [16]. The ability of PU.1 to bind to and repress GATA-1 transcriptional activity accounts for some of its functions in erythroid progenitors. However, PU.1 is also a DNA binding protein, with a number of well-established gene targets that it regulates in myeloid cells and B-cells [17]–[20]. Therefore, PU.1 might also regulate important genes in erythroid progenitors. Several examples of PU.1 gene targets with effects on erythroid cells have been described recently [11], [21], [22]. However, it is not known how many genes PU.1 controls in erythroid cells. Here we report that PU.1 does indeed direct an extensive transcriptional network in immature erythroid cells, a network consisting of pathways that are important for the growth and survival of erythroid progenitors. Moreover, we find that several of these pathways are also regulated by PU.1 in other hematopoietic lineages, suggesting that PU.1 has overlapping functions in several hematopoietic lineages.

## Results

### Genome-wide occupancy of PU.1 is highly similar in normal and leukemic erythroid cells

**Figure 1:**
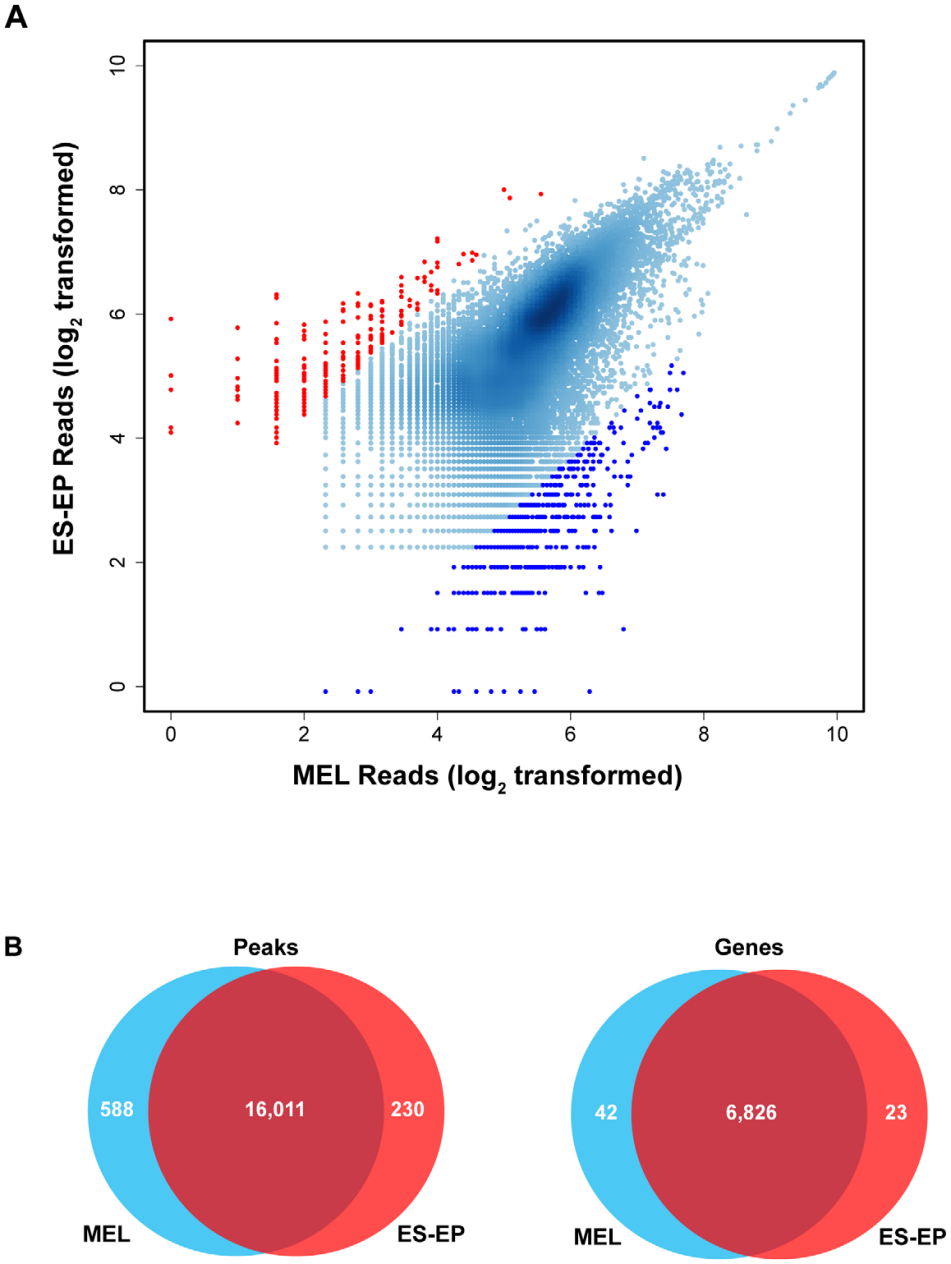
Genome-wide binding of PU.1 is very similar in ES-EP and MEL cells. (A) ChIP-Seq peaks were identified as described in Materials and Methods. A point represents the log_2_ of the number of sequence reads from ES-EP and MEL cells contained in a peak. Each peak was assigned to one of two categories based on the ratio of the number of reads in the two cell types. Peaks within a ratio ≤5 are classified as shared between both cell types (represented in light blue), whereas peaks with a ratio >5 are enriched in a cell specific manner (represented in red for ES-EP or dark blue for MEL cells). (B) Venn diagrams illustrate the number of peaks (left) or genes with a peak within 2 kb of TSSs (right) in ES-EP and MEL cells (colors as A).

**Figure 2:**
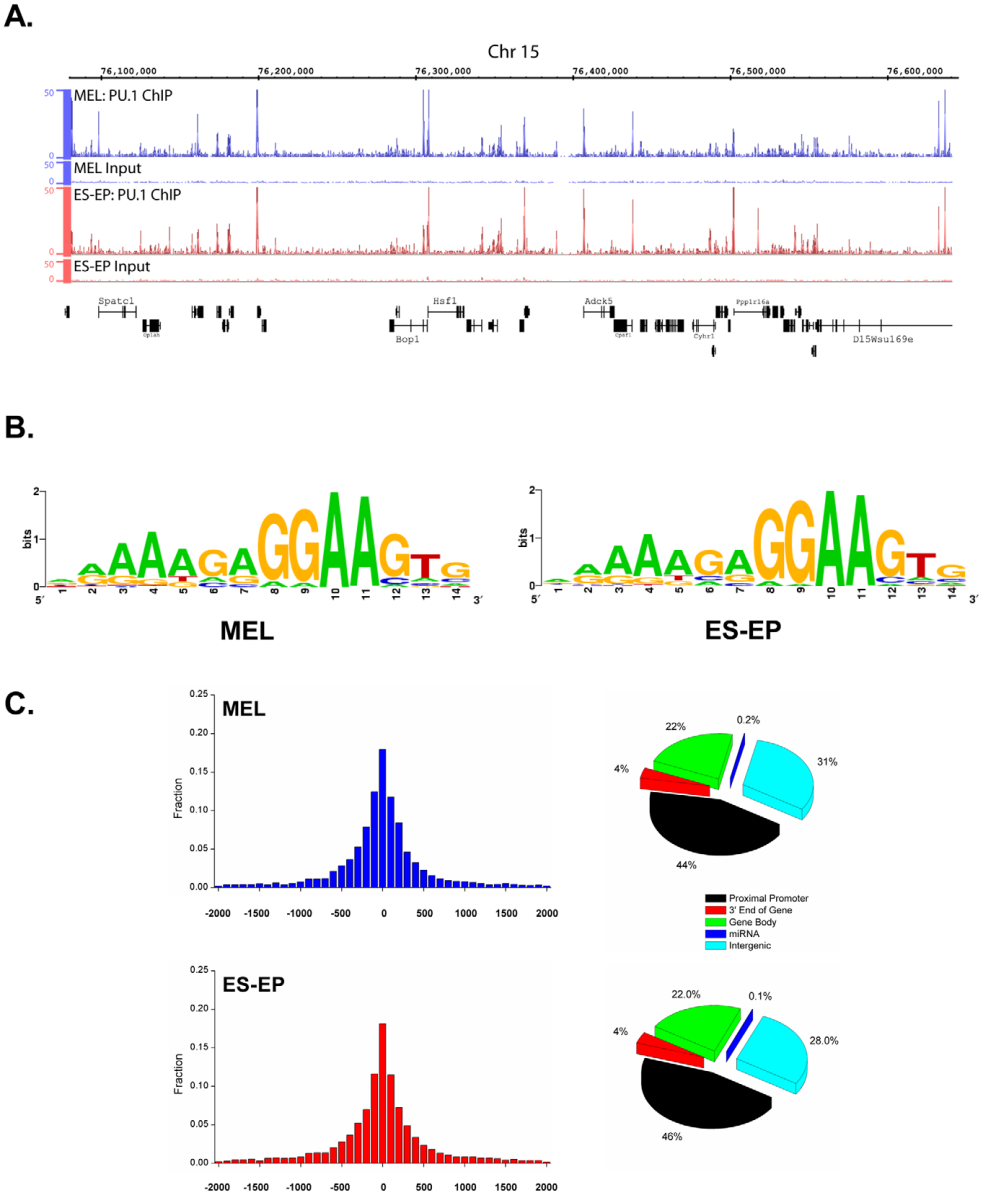
Properties of the PU.1 ChIP-Seq peaks in ES-EP and MEL cells. (A) Sample signal tracks of PU.1 ChIP-Seq data from MEL cells and ES-EP are shown for an ∼500 kb region of chromosome 15 in the Integrated Genome Browser (IGB) (Affymetrix), with the y-axis representing the number of reads. Input DNA controls are also shown for both cell types. (B) Sequence logos for the enriched motifs within PU.1 ChIP-Seq peaks from MEL (left) and ES-EP (right) cells, derived from MEME motif analysis (see Materials and Methods). (C) The distance between each PU.1 ChIP-Seq peak and the TSS within 2 kb was computed and the results are binned and plotted for MEL (top left) and ES-EP (bottom left). Peaks were further annotated by their genomic locations with respect to current gene annotation (right) and classified as proximal promoter (+/−2 kb of TSS), 3′ end of gene (+/−2 kb of TES), gene body (between +2 kb of TSS and −2 kb of TES), miRNA promoters, or otherwise intergenic regions.

As a first step in identifying the transcriptional network controlled by PU.1 in immature erythroid cells, we performed ChIP-Seq in normal proliferating erythroid progenitors derived from embryonic stem cells (ES-EP) [23] and leukemic erythroblasts (MEL cells). We obtained a total of 13,416,531 and 12,710,420 uniquely mapped reads in ES-EP and MEL cells, respectively. Using two peak calling programs, cisGenome and spp [24], [25], we identified a total of 16,241 peaks of PU.1 occupancy in ES-EP and 16,599 peaks in MEL cells. We also compared the number of reads in a given peak in MEL cells and ES-EP and then assigned a peak as present in both cell types (≤5 fold difference in the number of reads) or enriched in one cell type (>5 fold difference) (Figure 1A). With this classification scheme, we identified 16,011 peaks that are shared between the two cell types and 230 and 588 peaks that are enriched in ES-EP and MEL cells, respectively (Figure 1B left). Strikingly, more than 95% of the peaks are shared between the two cell types (Figure 1B left). Statistical analysis of the data presented in Figure 1A also revealed a strong similarity between PU.1 binding in the two cell types as evidenced by a correlation coefficient of 0.800 (p-value<2.2×10^−16^). This similarity is even visually evident from examination of the signal tracks of 500 kb windows, such as the one displayed in Figure 2A. We used qChIP to verify that some of the rare loci enriched in one cell type are indeed differentially occupied in the two cell types (Figure S1). Overall, our results indicate that the binding patterns of PU.1 are highly similar in normal and leukemic erythroid cells.

Since PU.1 occupies a surprisingly large number (>16,000) of sites in ES-EP and MEL cells (Figure 1B), it was of interest to determine whether association of PU.1 with such sites is directed by a PU.1 consensus binding sequence or whether many of these sites do not contain such a sequence, which would suggest that PU.1 occupancy is likely due to protein-protein interactions with other transcription factors. Analysis of the ChIP-Seq peaks in ES-EP and MEL cells with the MEME program [26] generated the position-weighted matrices shown in Figure 2B. The matrices, which are nearly identical for the two cell types, contain a purine-rich core sequence, GGAA, corresponding to the previously described core sequence present in PU.1 binding sites [27], [28] and matching the known binding sequence of the Ets family of transcription factors [19]. However, our analysis also identified a bias for an AAAGA sequence upstream of the purine-rich core, consistent with a recent report [17]. Of note, 13 of 14 positions in the matrices have a strong bias for purine residues. We found that 74% of peaks shared in ES-EP and MEL cells contain the consensus motif shown in Figure 2B, indicating that occupancy by PU.1 at the majority of observed sites is due to direct interaction between PU.1 and DNA. MEME analysis of the 26% of peaks that did not contain a PU.1 consensus motif showed enrichment for highly repetitive sequences that did not match any known transcription factor binding sites in the TRANSFAC database (data not shown).

### PU.1 binds near transcription start sites and regulates a large number of genes in immature erythroid cells

To better understand the network of genes that are potentially regulated by PU.1 in immature erythroid cells, we sought to associate peaks with genes. We determined that 6,826 of the PU.1 occupied sites shared by ES-EP and MEL cells lie within 2 kb of the transcription start site (TSS) of known genes (referred to as the proximal promoter) (Figure 1B right). Moreover, we find that the greatest concentration of peaks within the proximal promoter is found at the TSS in both cell types (Figure 2C left). Further analysis of the genome-wide distribution of peaks showed that ∼70% of peaks are found either within a gene structure or in the flanking 2 kb region, whereas ∼30% of peaks are located in intergenic regions, suggesting the main mechanism of PU.1-mediated transcriptional regulation is through short-range interactions with the transcriptional machinery (Figure 2C right). This is in contrast to recent reports on the distribution of PU.1 in other hematopoietic cell types [17], [18], [29] and our unpublished data in macrophages and B-cells (see Discussion). Interestingly, a larger percentage (∼40%) of shared peaks lie within proximal promoters, whereas a much smaller percentage (∼10%) of the peaks enriched in either ES-EP or MEL cells exhibit this characteristic (Figure S2A). Moreover, MEME analysis showed that the weighted matrices of the enriched peaks are slightly different from the weighted matrices from all peaks (compare Figure 2B and Figure S2B).

**Figure 3:**
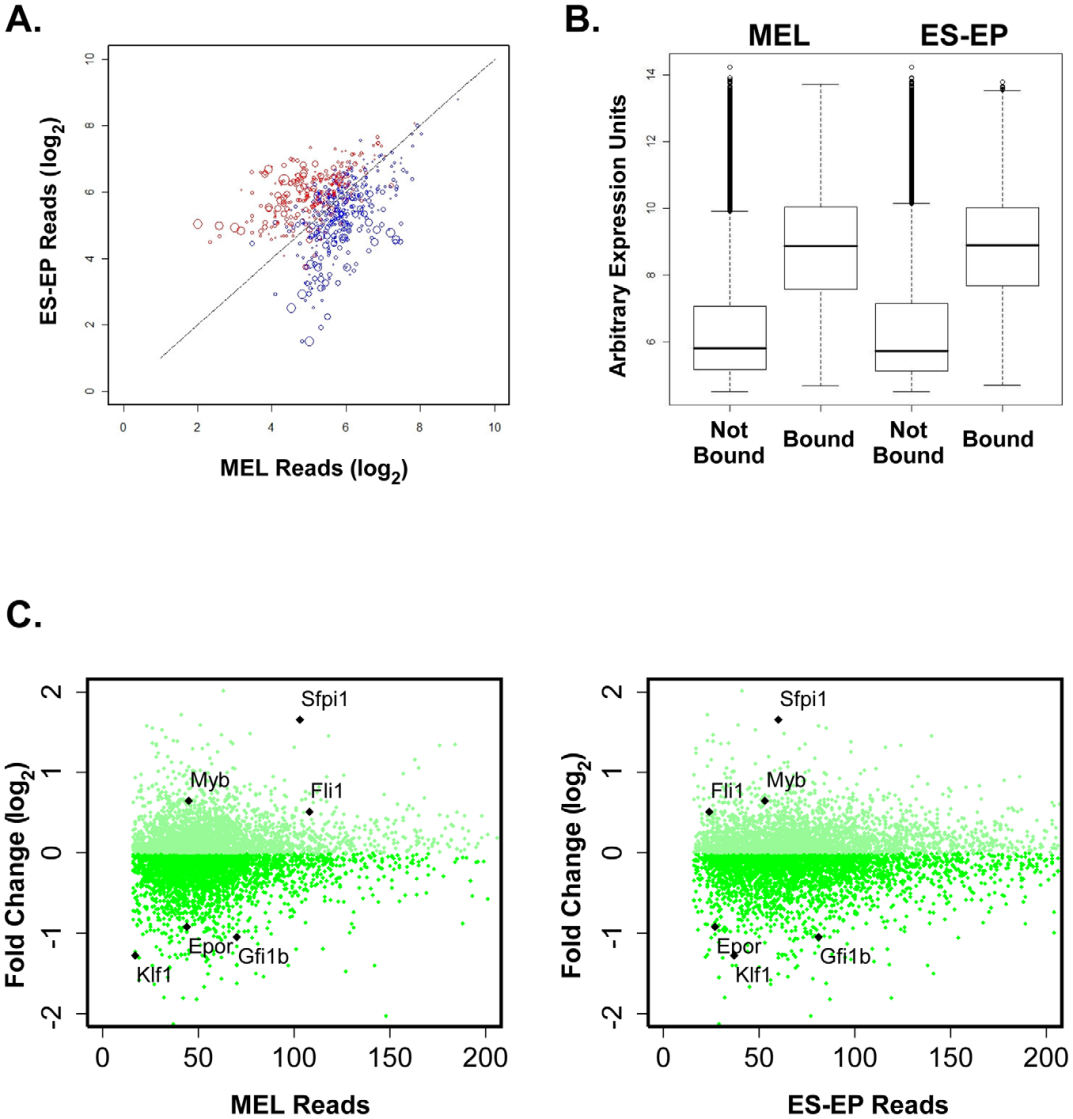
PU.1-dependent gene expression in erythroid cells. (A) PU.1 target genes with a 2-fold expression difference (as measured by Affymetrix gene array) between MEL and ES-EP cells were identified. The log_2_ of the number of PU.1 ChIP-Seq reads within peaks of these genes' promoters are computed and plotted here. Blue circles represent genes for which expression is higher in MEL cells, while red circles represent genes for which expression is higher in ES-EP. The diameter of each circle is proportional to the degree of difference in gene expression between the two cell types. (B) The expression levels of genes with and without PU.1 bound at proximal promoters in MEL and ES-EP are shown as boxplots, with the lower and upper sides of the box representing the lower and upper quartiles, respectively, and the line in the box representing the median expression value. (C) The log_2_ratio of the fold change in gene expression from early erythroid progenitors derived from wild-type embryos compared with PU.1 low embryos for genes bound by PU.1 in MEL (left) or ES-EP (right) are plotted as a function of the number of reads associated with the peak(s) corresponding to each gene. The positions of the values for Klf1, EpoR, Gfi1b, Myb, Fli1, and Sfpi1 in the plots are labeled.

Since PU.1 was found to bind to many genes, it was important to understand the functional consequences of PU.1 occupancy in immature erythroid cells. Therefore, we correlated the PU.1 ChIP-Seq data with two types of mRNA profiling in these cells. First, we compared the transcriptomes of ES-EP and MEL cells. We found that the mRNA profiles of the two cell types are quite similar, despite the differences in their phenotypic properties (Figure S3). Nevertheless, we identified 758 genes that are occupied by PU.1 near their TSS (PU.1 target genes) and that are differentially expressed by 2-fold or more between the two cell types (440 genes upregulated in ES-EP and 318 genes upregulated in MEL). For these genes, we compared their PU.1 occupancy (represented as the number of reads in the PU.1 peak(s) near their TSS) with their relative levels of expression in the two cell types. This analysis showed that genes differentially expressed ≥2-fold more in one cell type have higher PU.1 occupancy in that cell type (Figure 3A). This result suggests that, for genes differentially expressed between the two cell types, PU.1 occupancy often leads to upregulation of the gene's expression in that cell type, and hence PU.1 is acting on such genes primarily as a transcriptional activator. Consistent with this view, we also find in both ES-EP and MEL cells that the average level of expression of PU.1 target genes is significantly higher than that of non-PU.1 target genes (Figure 3B).

To determine how PU.1 affects the expression of its target genes, we combined the ChIP-Seq data with comparative gene expression analysis of fetal liver erythroid progenitors from wild-type E13.5–14.0 embryos and embryos that have a deletion in an Upstream Regulatory Element (URE) at the *Sfpi1* locus encoding PU.1, resulting in reduced PU.1 expression [30]. mRNA profiling was performed on very early committed erythroid progenitors (CD71^med^ TER119^low^) isolated by flow cytometry [31]. The PU.1 mRNA level was found to be reduced by about 70% in the PU.1 low erythroid progenitors relative to wild-type progenitors (Figure 3C), similar to reports in other cell types [30], [32], [33]. The gene expression analysis revealed that 617 genes are up regulated and 836 genes are down regulated by PU.1 at least 1.5 fold in these cells. Of these 1453 genes exhibiting PU.1 dependent expression in erythroid progenitors, 504 genes (35%) have PU.1 bound within 2 kb of their TSS. Therefore, 7.4% (504/6826) of PU.1 bound genes are regulated by PU.1. Interestingly, when we correlated the gene expression changes in erythroid cells to the level of PU.1 occupancy, we found that genes exhibiting the strongest PU.1-dependent gene expression changes have a lower level of PU.1 binding (Figure 3C), a phenomenon that needs to be further explored. These findings reveal that a large number of genes are bound and regulated in a PU.1-dependent manner in erythroid progenitors.

### PU.1 regulates genes involved in controlling erythroid differentiation

**Figure 4:**
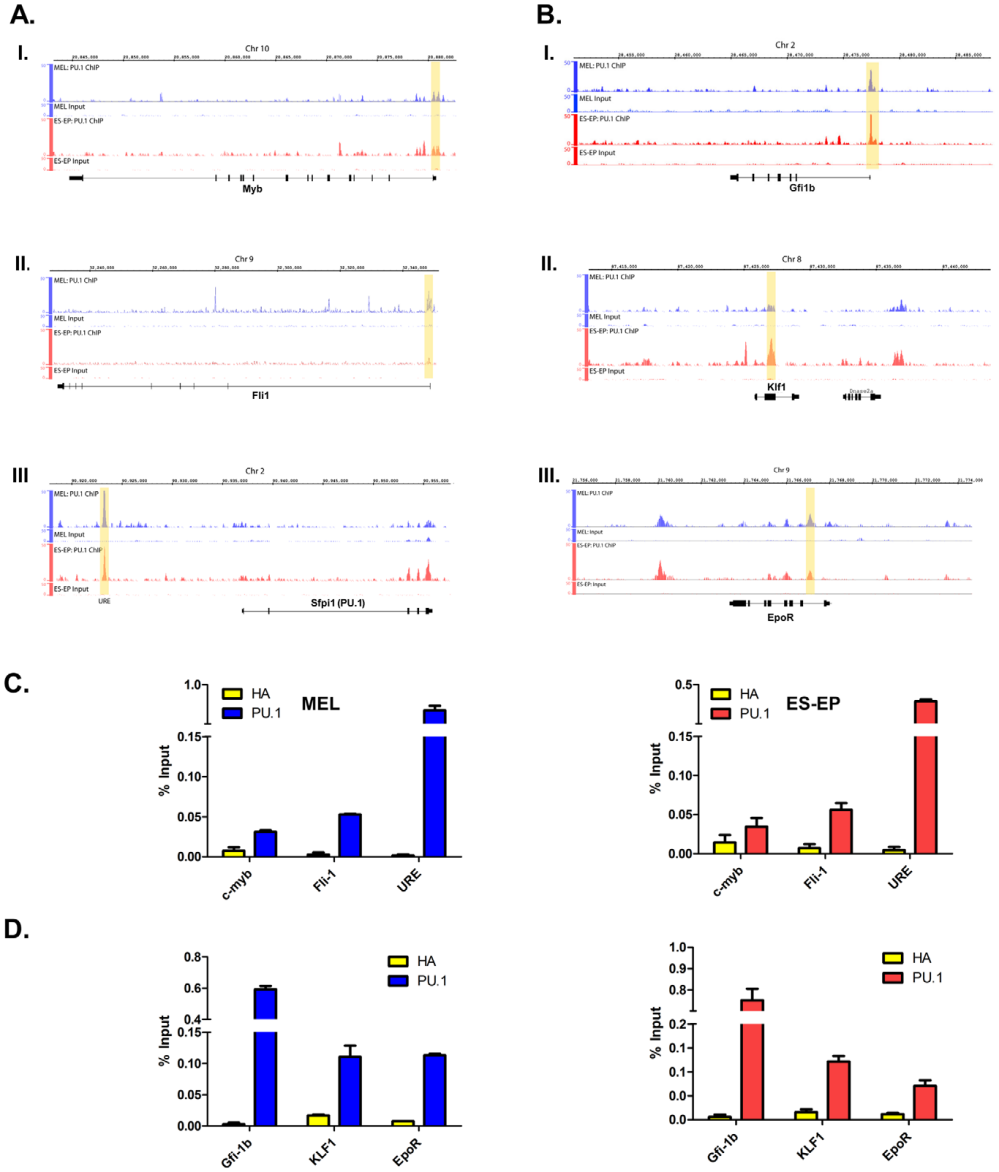
PU.1 regulates genes involved in controlling erythroid differentiation. (A) and (B) Signal tracks of PU.1 ChIP-Seq data from MEL cells (blue) and ES-EP (red) in the vicinity of the genes depicted schematically (black) using IGB. Myb, Fli1, Gfi1b, and EpoR are transcribed from the negative strand (right to left), whereas Sfpi1 (PU.1) and Klf1 are expressed in the sense direction (left to right). Input DNA controls are also shown for both cell types. (C) and (D) qChIP validations of PU.1 occupancy at the regions highlighted in yellow in (A) and (B). qChIP was performed as described in Materials and Methods with chromatin from MEL cells (left) and ES-EP (right) with primers described in Table S2. A HA antibody was used as an isotype control. Standard deviations were calculated from triplicate PCR reactions. Similar results were obtained with at least two independent chromatin preparations.

Besides PU.1, a number of other factors have been shown to be involved in regulating erythroid differentiation and erythroid-specific gene expression. For example, the Ets protein Fli-1 [34], [35] and c-Myb [36], [37], like PU.1, inhibit erythroid differentiation, whereas Gfi-1b [38], the erythropoietin receptor (EpoR) [39], and the erythroid kruppel-like factor Klf1 [40], [41] promote erythroid differentiation. Comparative gene expression analysis in wild-type and PU.1 low erythroid progenitors revealed that c-myb and Fli-1 are upregulated by PU.1, whereas Gfi-1b, Klf1, and EpoR are significantly downregulated (Figure 3C). Furthermore, our ChIP-Seq data shows PU.1 occupancy either very near to the TSS and/or within the transcribed region of each of these genes in both normal ES-EP and MEL cells (Figure 4A and 4B). These data were confirmed by qChIP in both cell types (Figure 4C and 4D). Interestingly, we observed very high levels of PU.1 occupancy in erythroid cells at the Upstream Regulatory Element (URE) lying ∼14 kb upstream of the PU.1 (Sfpi1) gene itself (Figure 4AIII and 4C). The URE has been shown to have a strong positive effect on PU.1 expression in myeloid cells [30], [42]. Indeed, deletion of the URE element resulted in about a 70% reduction of PU.1 in early fetal liver erythroid progenitors, similar to other cell types, suggesting PU.1 upregulates its own expression in immature erythroid cells (Figure 3C, [30], [32], [33]. The direct positive and negative effects of PU.1 on expression of the aforementioned genes fit well with their observed roles in erythroid differentiation, further strengthening the idea that PU.1 controls the differentiation decision in erythroid cells.

### PU.1 regulates several pathways involved in controlling the growth and survival of immature erythroid cells

To further understand the categories of genes and biological pathways regulated by PU.1 in erythroid cells, we analyzed the ChIP-Seq PU.1 targets with Ingenuity Pathway Analysis (IPA) software. Table S1 shows the ten most significantly over-represented categories of molecular and cellular functions that are represented in the PU.1 target genes in both ES-EP and MEL cells. Interestingly, the two categories at the top are gene expression and cell cycle. To validate that PU.1 does indeed bind within the proximal promoter of genes in these two categories, we carried out qChIP experiments on a total of 32 genes, in both ES-EP and MEL cells. 84% and 94% of the 32 genes in each cell type, respectively, were validated for PU.1 occupancy by qChIP (Figure 4, Figure S1, and Figure S4).

**Figure 5:**
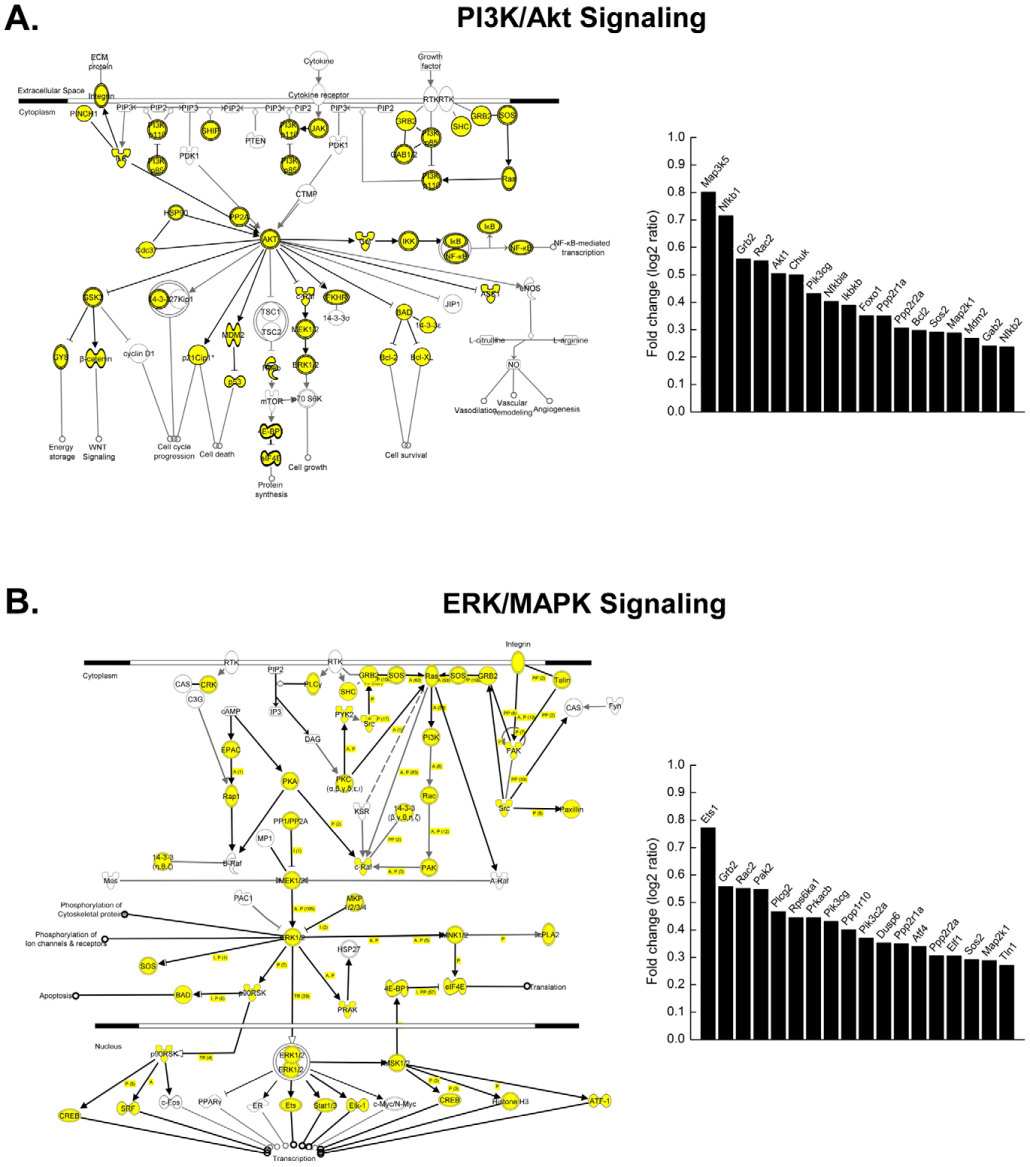
PU.1 regulates many genes in the PI3K/Akt and ERK/MAPK signaling pathways. (A) and (B) IPA analysis was performed as described in Materials and Methods on PU.1 target genes in both ES-EP and MEL cells. The PI3K/Akt and ERK/MAPK signaling pathways were identified as being significantly over-represented among those genes. The left part of each panel depicts all of the components of the indicated IPA pathway. Components denoted with a filled yellow circle indicate that the gene for that component is associated with a PU.1 ChIP-Seq peak in both ES-EP and MEL cells. To the right of each pathway is shown the results of gene expression analysis of the indicated genes in early erythroid progenitors derived from wild-type embryos relative to PU.1 low embryos.

IPA analysis also revealed several important cellular pathways that are regulated by PU.1 in erythroid cells. For example, 58% (79/137) of genes involved in the PI3K/Akt signaling pathway are occupied by PU.1 in ES-EP and MEL cells (Figure 5A). Gene expression analysis of wild-type and PU.1 low erythroid progenitors indicates that many genes that stimulate this pathway are positively regulated by PU.1 (Figure 5A). Another pathway found to be regulated by PU.1 in erythroid cells is the ERK/MAPK signaling pathway. 48% (92/192) of the genes in this IPA pathway are occupied by PU.1 (Figure 5B). As with the PI3K signaling pathway, gene expression analysis shows that PU.1 upregulates many genes that stimulate ERK/MAPK signaling (Figure 5B). IPA analysis also showed that PU.1 regulates the Jak/Stat signaling pathway. 67% (43/64) of the genes in this pathway are occupied by PU.1, and many of these genes are upregulated by PU.1 (data not shown). As discussed below, the PI3K/Akt, ERK/MAPK, and Jak/Stat signaling pathways have all been shown to play important roles in erythroid cell proliferation, survival and differentiation. Therefore, these results indicate that PU.1 regulates many genes and pathways that are crucial for erythroid cell function.

### PU.1 levels regulate the number of early erythroid committed cells *in vivo*

**Figure 6:**
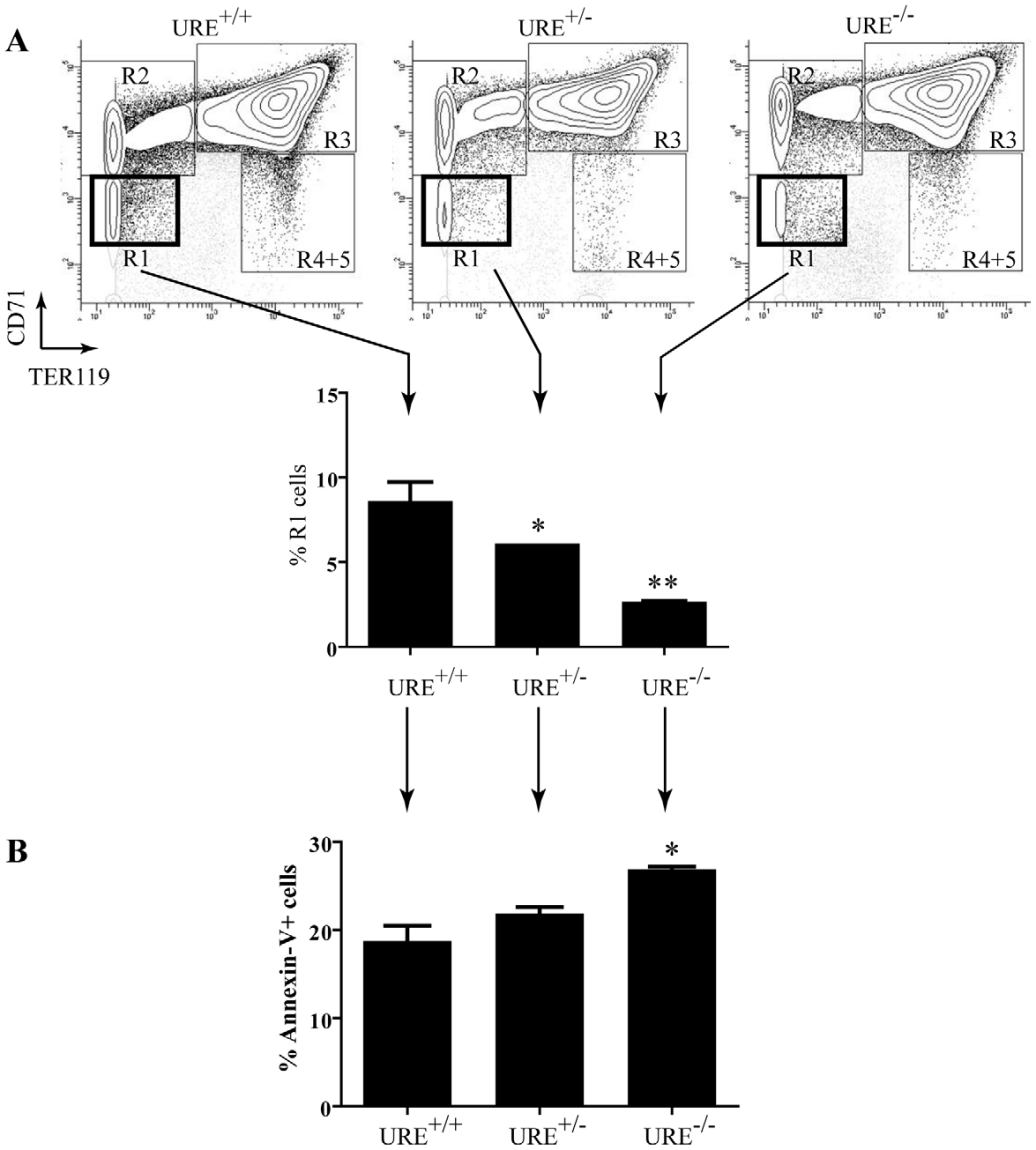
Reduction in early erythroid progenitors in PU.1 depleted embryos. (A) Fetal livers were isolated from E13.5–14 day old embryos from WT (URE^+/+^), heterozygous (URE^+/−^), and homozygous (URE^−/−^) animals. Distinct erythroid populations were identified by staining with TER119 and CD71, with representative stainings shown. The percent of R1 cells present from two animals from each genotype was averaged and is displayed as a bar graph. * t-test<0.05, ** t-test<0.01 compared to URE^+/+^. (B) Apoptosis was analyzed by Annexin-V staining of R1 cells. The percentage of Annexin-V positive cells is shown as an average of two animals from each genotype. *t-test<0.05 compared to URE^+/+^.

The foregoing results indicate that PU.1 regulates several pathways that control the proliferation, survival and differentiation of erythroid cells. Previous studies reported that *ex vivo* cultures of PU.1-depleted fetal liver erythroblasts [10] or preleukemic erythroblasts [43] exhibit defects in proliferation and increased cell death. To determine whether PU.1 levels regulate the number of erythroid progenitors *in vivo* and the precise stage of its effects, we analyzed the distribution of five distinct populations of erythroid cells in fetal livers of mutant PU.1 low embryos and wild-type littermate embryos [30]. The five populations were identified by flow cytometry analysis for erythroid-specific TER119 and the transferrin receptor (CD71), representing populations of progressively more mature stages of erythroid cells from the earliest erythroid committed cells (R1 - CD71^med^ TER119^low^) to late *orthochromatophilic* erythroblasts and reticulocytes (R5 - CD71^med^ TER119^high^), as described previously [31]. We observed a dose-dependent reduction in the percentage of R1 cells from fetal livers of heterozygous and homozygous mutant embryos compared to wild-type littermate embryos (Figure 6A). Whereas, on average 8.6% of the fetal-liver of wild-type embryos are comprised of R1 cells, this compartment constitutes only 6.1% (t-test p-value 0.045 compared to wild-type) and 2.7% (t-test p-value 0.009 compared to wild-type) in heterozygous and homozygous mutant embryos, respectively. Given that PU.1 promotes several critical pathways involved in promoting survival, we hypothesized that the reduction of the R1 population may be due to increased apoptosis of these cells. Indeed, Annexin-V staining showed that the reduction in R1 cells is due, at least in part, to increased cell death in this population (Figure 6B). These results are consistent with a role for PU.1 in promoting widely utilized pathways for maintaining cell survival in very early erythroid committed cells *in vivo*.

### PU.1 may cooperate with E2Fs and other transcription factors in erythroid cells

Lineage determining transcription factors, like PU.1, are thought to function together with other general and cell type-specific transcription factors to modulate gene expression [17], [29]. To identify candidate transcription factors that may cooperate with PU.1 in immature erythroid cells, we scanned the DNA sequences within the shared PU.1 peaks in ES-EP and MEL cells for potential transcription factor binding sites using the TRANSFAC database (see Materials and Methods). The analysis revealed that there is a non-random, statistically significant (p-value<10^−5^) association of PU.1 occupied sites with consensus binding-site sequences for a number of well-studied and less well-studied transcription factors (Figure S5A). For example, consistent with a potential role for PU.1 in cell cycle regulation, consensus binding-site sequences for E2F factors are found within the PU.1 ChIP-Seq peaks. Consensus binding site sequences for another factor, ETF, which has been implicated in control of organ size via effects on cell proliferation [44], are also enriched within the PU.1 ChIP-Seq peaks.

To determine if E2F factors are indeed associated with PU.1-occupied genes in erythroid cells, we performed qChIP studies of E2F2 and E2F4, two factors that have been reported to be involved in erythropoiesis [45]–[48]. One or both factors were found to bind near the sites occupied by PU.1 in the promoters of a number of genes in MEL cells (Figure S5B, S5C, and Figure S4). Occupancy of PU.1 and E2F2 was more prevalent than PU.1 and E2F4. Interestingly, both E2F2 and E2F4 are present, along with PU.1, at the PU.1 URE itself, while E2F4 and PU.1 are bound to the PU.1 promoter region. Although further studies are needed to understand how E2Fs may cooperate with PU.1 to regulate gene expression in erythroid and possibly other hematopoietic cells, these results support the view that lineage-specific transcription factors work in concert with widely expressed factors.

### PU.1 regulates many of the same genes and pathways in immature erythroid cells and other hematopoietic cells

**Figure 7:**
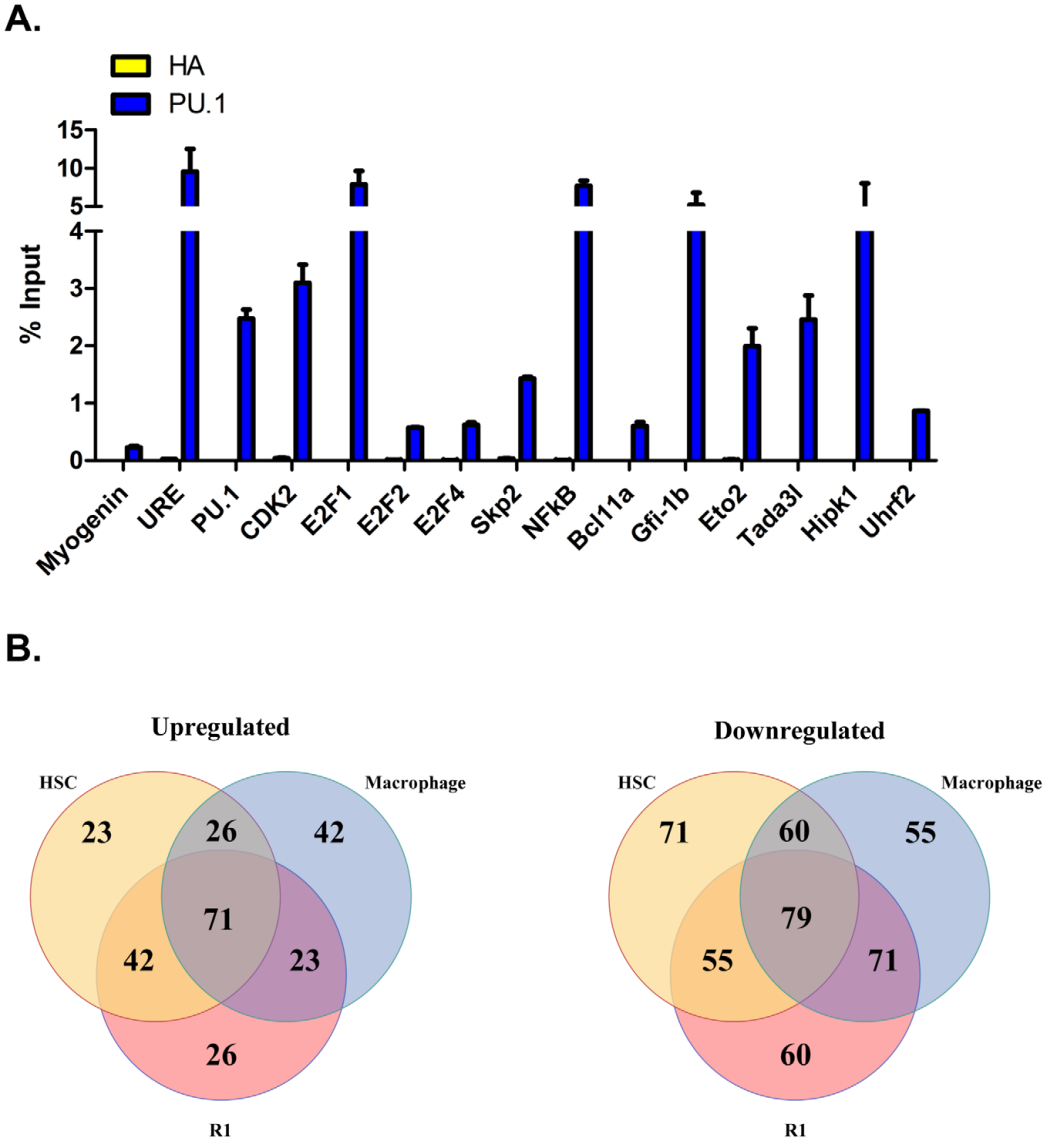
PU.1 occupies many of the same gene targets in myeloid and erythroid cells, and PU.1-dependent gene regulation is similar in HSC, myeloid, and erythroid cells. (A) qChIP was performed for PU.1 as described in Materials and Methods with chromatin from 32D cells using primers described in Table S2. An HA antibody was used as an isotype control. The same primers were used to show that PU.1 binds close to these genes in MEL cells and ES-EP (Figure S5). The myogenin gene serves as a negative control. Standard deviations were calculated from triplicate PCR reactions. Similar results were obtained with at least two independent chromatin preparations. (B) Expression data from PU.1^−/−^ cells stimulated to differentiate into macrophages by the restoration of PU.1 [49] and HSC from PU.1 knockdown mice [33] were used to compare with PU.1 dependent gene regulation in early erythroid progenitors from PU.1 knockdown mice. Of the PU.1 bound genes in MEL and ES-EP that displayed a ≥1.5-fold change in expression, 308 were upregulated and 209 were downregulated. 162 and 265 of these PU.1 dependent genes were annotated in the other 2 datasets. Venn diagrams display the comparisons between the three datasets of these upregulated genes (left) and downregulated genes (right).

Since PU.1 is an established transcriptional regulator in myeloid cells, we sought to determine if genes that we observed to be regulated by PU.1 in erythroid cells are also regulated by PU.1 in myeloid cells. We performed qChIP analysis in a myeloid cell line (32D) at 15 gene targets bound by PU.1 in erythroid cells. We found PU.1 bound 100% of these genes in myeloid cells (Figure 7A), consistent with a recent study in which 80% of PU.1 peaks lying close to TSS were found to overlap in macrophages and B cells [17]. Since we also observed that PU.1 regulates many genes in the PI3K/Akt and ERK/MAPK pathways in erythroid cells (Figure 5), we compared our findings with a published gene expression analysis of PU.1 null cells undergoing differentiation to macrophages in response to restoration of PU.1 [49]. We found that, just as we observed in erythroid cells, many of the genes in these two pathways are upregulated by PU.1 in macrophages (Figure S6). Additionally, we also compared all of the genes regulated by PU.1 in early erythroid committed (R1) cells with the macrophage gene expression data, as well as with a gene expression analysis of HSC from PU.1 knockdown mice [33]. Of the genes that we found to be occupied and upregulated by PU.1 at least 1.2-fold in R1 cells, and also annotated in the two other data sets, 58% (94/162) are also upregulated in a PU.1 dependent-manner during macrophage differentiation and 70% (113/162) are similarly regulated in HSC (Figure 7B left). Similarly, of the genes that we found to be bound and repressed by PU.1 in erythroid cells and also represented in the other data sets, 57% (150/265) are also downregulated by PU.1 in macrophages and 51% (134/265) are repressed in a PU.1-dependent manner in HSC (Figure 7B right). We conclude that PU.1 regulates many of the same genes and pathways in immature erythroid cells, myeloid cells and HSC (see Discussion).

## Discussion

### The different roles of PU.1 in immature erythroid cells

PU.1 is a master regulatory transcription factor that plays an essential role in the development of the myeloid and B-cell lineages [4], [5]. However, several lines of evidence suggest that PU.1 also plays important roles in erythropoiesis (see Introduction and Figure 6). Some of its effects are likely attributable to its ability to bind to and repress GATA-1 [3], [7], [50], but it is also very important to understand the global gene expression program controlled by PU.1 in immature erythroid cells. To investigate this program, we first determined the genome-wide binding patterns of PU.1 in normal erythroid progenitors and leukemic erythroblasts. We found that PU.1 occupancy is highly similar in both cell types and that PU.1 binds in close proximity to a large number of genes (Figure 1). Unexpectedly, PU.1 appears to occupy more sites (>16,000) in immature erythroid cells than three essential red blood cell transcription factors, including GATA-1 (reported to occupy between 4,000–14,400 sites) [51]–[54], SCL (<4,500 sites) [55], and Klf1 (<2,000 sites) [56]. Although some of these differences may be due to the use of different peak calling methods or other technical issues such as the use of different antibodies and different cells, our unpublished results on GATA-1 occupancy using the same methods and the same cells lead to a similar conclusion (unpublished data). Interestingly, the genomic distributions of PU.1 in erythroid cells are also dramatically different from that reported for GATA-1 and Klf1. Whereas we found ∼40% of PU.1 bound sites are within 2 kb of TSS (Figure 2), only ∼16% of Klf1 bound sites lie within 1 kb of TSS [56], and only ∼13% of GATA-1 sites are found within −10 kb of TSS [51], consistent with our unpublished results indicating that only 9% of GATA-1 bound sites are within 2 kb of TSS in ES-EP (unpublished data). These results suggest that PU.1 may act independently of these factors, at least at a subset of genes in erythroid progenitors.

Recent work demonstrates that PU.1 directly regulates certain genes that can themselves affect erythroid differentiation. For example, we reported that the CDK6 gene is directly upregulated by PU.1 [11]. Furthermore, we also showed that CDK6, like PU.1, is rapidly downregulated as erythroid cells enter terminal differentiation and that constitutive expression of CDK6 blocks erythroid differentiation [57]. Likewise, Elf-1, another member of the Ets transcription factor family, was shown to be directly upregulated by PU.1 and to negatively regulate erythroid differentiation [22]. Here we report that PU.1 binds in close proximity to the genes that encode Fli1, c-myb, and PU.1 itself (Figure 4). We also showed that PU.1 upregulates the expression of each of these genes (Figure 3C). Like PU.1, Fli1 and c-myb have been shown to block erythroid differentiation [34], [36], [37], [58]–[60]. On the other hand, we also found that PU.1 binds in close proximity to and inhibits expression of genes that promote erythroid differentiation, such as Gfi-1b, EpoR, and Klf1 (Figure 3C and Figure 4) [38]–[41]. Thus, our results show that PU.1 regulates the expression of genes that both inhibit and promote erythroid differentiation.

However, the results reported here also reveal that PU.1 has a much broader role in these cells than simply regulating several factors that control erythroid differentiation. Indeed, the ChIP-Seq and transcriptome analyses show that PU.1 regulates many genes involved in several signaling pathways that are critical for erythropoiesis. These pathways include the PI3K/Akt, ERK/MAPK, and Jak/Stat pathways (Figure 5 and data not shown), all of which have been implicated in survival and proliferation of erythroid progenitors [61]–[64]. Interestingly, the PI3K/Akt and Jak/Stat pathways regulate PU.1 levels in erythroid cells [65], [66]. Taken together, these findings suggest the existence of a feedback loop in which PU.1 controls the expression of many genes in these crucial signaling pathways and these pathways upregulate the expression of PU.1. Our finding that mice with ∼30% of normal PU.1 levels exhibit a marked reduction in the number of the earliest committed fetal liver erythroid progenitors (Figure 6) is consistent with an important role for PU.1 in regulating these pathways *in vivo*. Thus, our studies further advance the concept of master regulatory transcription factors regulating both lineage-specific genes and more widely expressed genes.

### Overlapping functions of PU.1 in several hematopoietic lineages

Several recent papers reported the genome-wide binding patterns of PU.1 in macrophages [17], [18], [20], neutrophilic precursors [19], B-cells [17], and multipotent progenitors cells [29]. Interestingly, the genomic distributions of PU.1 in these cells are quite different from what we observed in erythroid cells. For example, whereas we found that ∼40% of PU.1 occupied sites map within 2 kb of TSS (the overwhelming majority of these being bound within 500 bp of TSS) (Figure 2), one study found that only 12% and 18% of PU.1 binding sites in macrophages and B-cells, respectively, were within 500 bp of TSS [17], and another study in macrophages found only 20% of such sites to be within 2.5 kb of TSS [18]. Similarly, only 23% of PU.1 peaks mapped within 1 kb of TSS in multipotential hematopoietic progenitor cells [29]. It is likely that part of the reason for these differences is the larger number of PU.1 occupied sites in the other cell types compared with erythroid cells. In fact, comparison of ChIP-Seq data for PU.1 in macrophages and B-cells that we have generated, which resembles published data, shows that most peaks observed in the erythroid cells are also present in macrophages and B-cells (unpublished data). Consistent with this observation, we found that 100% (15/15) of genes occupied by PU.1 in erythroid cells are also occupied by PU.1 in 32D cells (Figure 7). Furthermore, most of the genes targeted for regulation by PU.1 in erythroid cells are not lineage specific; rather they are genes that are widely expressed in many cell types. This observation begs the question: Does PU.1 control common sets of genes in the several hematopoietic lineages in which it is expressed? To answer this question, we made several different types of comparisons of PU.1 gene targets in erythroid cells, myeloid cells and HSC. For example, we observed that many PU.1 gene targets in the PI3K/Akt and ERK/MAPK pathways that we identified in erythroid cells are similarly regulated by PU.1 in macrophages (Figure 5 and Figure S6). We also found that the majority of genes that are occupied and either positively or negatively regulated in erythroid cells are similarly regulated in macrophages and HSC (Figure 7B). Taken together, these comparative analyses indicate a very significant overlap in PU.1-dependent gene regulation in erythroid cells and other hematopoietic cells.

Much evidence supports the view that PU.1 plays a major role in establishing cellular identity in B-cells and myeloid cells. Finding such an extensive overlap among the PU.1 gene targets in several related but quite distinct hematopoietic cell types raises the question as to how cellular identity is established by PU.1. We suggest that the answer lies, at least in part, in the differences in the levels of PU.1 in different hematopoietic cells. Myeloid cells have the highest level of PU.1, B-cells have an intermediate level [67], and immature erythroid cells have a much lower level [68], [69]. Overexpression of PU.1 in B-cells reprograms the cells to macrophages [70], similar to what has been reported in MEL cells [71], [72]. These observations, taken together with our finding that many widely expressed genes are occupied and similarly regulated by PU.1 in immature erythroid cells, myeloid cells and HSC (Figure 7 and Figure S6), suggest that PU.1 has both shared and lineage-specific functions in these lineages, depending on the level of the factor in each lineage. We suggest that even at low concentrations, PU.1 can promote ubiquitous cellular functions such as proliferation and survival. Indeed, PU.1 has been reported to promote proliferation of both erythroid progenitors [10], [43] and bone marrow derived macrophages [73]. However, higher concentrations of PU.1 are required to promote its lineage-specific functions [67], [70]–[72]. It will be interesting to determine whether this principle of overlapping gene networks regulated by a transcription factor in several related lineages, proposed here for PU.1, is applicable to master transcriptional regulators in other developmental systems.

## Materials and Methods

### Cell culture

MEL cells (clone DS19) stably expressing a GATA-1-estrogen receptor ligand-binding domain (ER) fusion protein (GATA-1/ER) was described previously [74]. ES-EP cells were cultured as previously described [23]. Further details can be found in Text S1.

### qChIP and ChIP-Seq

qChIP was performed as previously described [11]. ChIP for ChIP-Seq analysis was performed similarly but using 5×10^7^ cells and 60 µg of anti-PU.1 antiserum. A list of antibodies used in this study, along with further information on processing of ChIP-Seq samples can be found in Text S1. A summary of PU.1 ChIP-Seq data can be found in Dataset S1.

### ChIP-Seq data analysis

Uniquely mapped reads from ChIP-Seq data were mapped to the mouse genome (mm9). Details on peak calling and subsequent statistical analysis can be found in Text S1. ChIP-Seq data is deposited on GEO database under the accession number GSE21953.

### Gene expression data analysis

Total RNA was isolated from duplicate cultures of proliferating ES-EP and MEL cells or R1 cells sorted from two wild-type and URE^−/−^ animals using the RNeasy Kit (Qiagen) following the manufacturer's instructions. Total RNA was further processed by the AECOM microarray facility using the standard Affymetrix pipeline and hybridized to GeneChip Mouse Gene 1.0 ST (Affymetrix). This expression data can be accessed from the GEO database under the accession number GSE21953. Subsequent statistical analysis is described in Text S1.

### Motif enrichment analysis

For each PU.1 peak, we extracted a 500-bp sequence around the peak center (i.e., +/−250 bp) and used it for searching *de novo* motifs with the MEME software [26] and also known motifs in the TRANSFAC software suit (release 11) [75]. For motif analysis with TRANSFAC, we utilized the program MATCH in the TRANSFAC software package and applied the default parameters for minimizing the false positive rate (i.e., minFP option). As our background control, we ran MATCH against a randomly chosen set of 10,000 sequences (500-bp each). The enrichment of a known motif in PU.1 peaks was then calculated as the ratio of the motif occurrence frequency in PU.1 peaks and its corresponding frequency in background sequences. P-value was derived with a binomial test of the difference in motif frequency.

## Supporting Information

Dataset S1 PU.1 ChIP-Seq Summary. Summary of PU.1 ChIP-Seq data. File includes all peaks identified in ES-EP and MEL cells. Peaks that are within 2 kb of TSS are assigned with a RefSeq gene.
(2.05 MB XLS)

Figure S1 Comparisons of ChIP-Seq and qChIP data for loci differentially occupied by PU.1 in MEL cells and ES-EP. (A) Comparisons for loci exhibiting enriched occupancy of PU.1 in MEL cells. Panels 1–3 display signal tracks corresponding to 1) a gene-poor region on chromosome 15, 2) the region near the Mxi1 gene, 3) the region near the Dnmt3a gene. Panel 4 shows qChIP analyses near the peaks denoted with arrows in panels 1–3. A HA antibody was used as an isotype control. Sequences of qChIP primers are shown in Table S2. Standard deviations represent the errors from triplicate PCR reactions. Similar results were obtained with at least 2 independent chromatin preps. (B) As in (A) for loci exhibiting enriched occupancy of PU.1 in ES-EP. Panels 1–3 display signal tracks corresponding to 1) a gene-poor region on chromosome 8, 2) the region near the Mta3 gene, 3) the region near the Gpr149 gene.
(0.41 MB PDF)

Figure S2 Properties of PU.1 ChIP-Seq peaks enriched in MEL cells or ES-EP. (A) The percentage of PU.1 ChIP-Seq peaks lying within the indicated distance from the closest TSS was calculated separately for peaks shared by the two cell types (Overlap) or enriched in either MEL cells or ES-EP, as described in Materials and Methods. (B) The derived position-weighted matrices from MEME analyses of PU.1 ChIP-Seq peaks enriched in MEL cells (top) and ES-EP (bottom) are shown. The thymidine [T] and adenosine [A] residues that differ relative to matrices derived from MEME analyses of all PU.1 ChIP-Seq peaks in each cell type (Figure 2B) are highlighted.
(0.27 MB PDF)

Figure S3 High correlation of gene expression in MEL cells and ES-EP. Gene expression levels were obtained separately for MEL cells and ES-EP with the Affymetrix microarray platform. After data processing and normalization, the relative expression of genes in the two samples are shown here as a scatter plot with larger units representing higher expression. The Pearson's correlation coefficient and its statistical significance are shown at the top of the figure.
(0.77 MB PDF)

Figure S4 qChIP validation of PU.1 occupancy near genes involved in gene regulation and cell cycle regulation. (A) and (B) qChIP was performed as described in Materials and Methods with chromatin from MEL cells (top) and ES-EP (bottom) with primers described in Table S2. The genes analyzed represent examples of genes from the IPA gene expression (A) and cell cycle (B) categories that have PU.1 ChIP-Seq peaks within +/−2 kb of their TSS. Myogenin and β-HS2 serve as negative and positive controls, respectively. A HA antibody was used as an isotype control. Standard deviations were calculated from triplicate PCR reactions. Similar results were obtained with at least two independent chromatin preparations.
(0.16 MB PDF)

Figure S5 E2F factors occupy PU.1 target genes in MEL cells. (A) DNA sequences from PU.1 ChIP-Seq peaks within the proximal promoter in both ES-EP and MEL cells were analyzed using the TRANSFAC database as described in Materials and Methods. The names of the ten most significant observed transcription factor motifs (p-value<10^−5^) that were found in at least 10% of peaks and ≥2 fold enriched over the expected frequency are shown. The ratio between observed frequency and expected frequency is represented by a log_2_ratio. (B) and (C) qChIP analysis of E2F2 (B) and E2F4 (C) occupancy was performed as described in Materials and Methods at the indicated PU.1 target genes in MEL cells. A HA antibody was used as an isotype control. Standard deviations were calculated from triplicate PCR reactions. Similar results were obtained with at least two independent chromatin preparations. The myogenin locus serves as a negative control.
(0.16 MB PDF)

Figure S6 PU.1 promotes PI3K/Akt and ERK/MAPK signaling in macrophages. A previously published [49] gene expression analysis of PU.1^−/−^ cells induced to differentiate into macrophages was used to generate heatmaps depicting the response of the indicated genes in the PI3K/Akt and ERK/MAPK signaling pathways to the activation of PU.1. The genes shown in this analysis are the same as the genes found to be regulated by PU.1 in MEL cells (Figure 5).
(0.11 MB PDF)

Table S1 Results of Ingenuity Pathway Analysis of PU.1 Target Genes. Ingenuity Pathway Analysis was performed on genes with one or more PU.1 ChIP-Seq peaks within 2 kb of TSS in both MEL and ES-EP. The ten most significant molecular and cellular functions identified are listed in descending order of significance. The number of genes associated with a given function and bound by PU.1 is shown. p-values have been corrected for multiple testing using the Holm-Bonferroni method.
(0.01 MB DOCX)

Table S2 Primers used in this study.
(0.02 MB DOCX)

Text S1 Supplemental Materials and Methods.
(0.06 MB DOC)
