# Supplementary figures and images for: A Large Gene Network in Immature Erythroid Cells Is Controlled by the Myeloid and B Cell Transcriptional Regulator PU.1
Source: PLoS Genet. 2011 Jun 9;7(6):e1001392. doi: 10.1371/journal.pgen.1001392 (PMC3111485; doi:10.1371/journal.pgen.1001392)

Figure S1 (Wontakal et. al.)

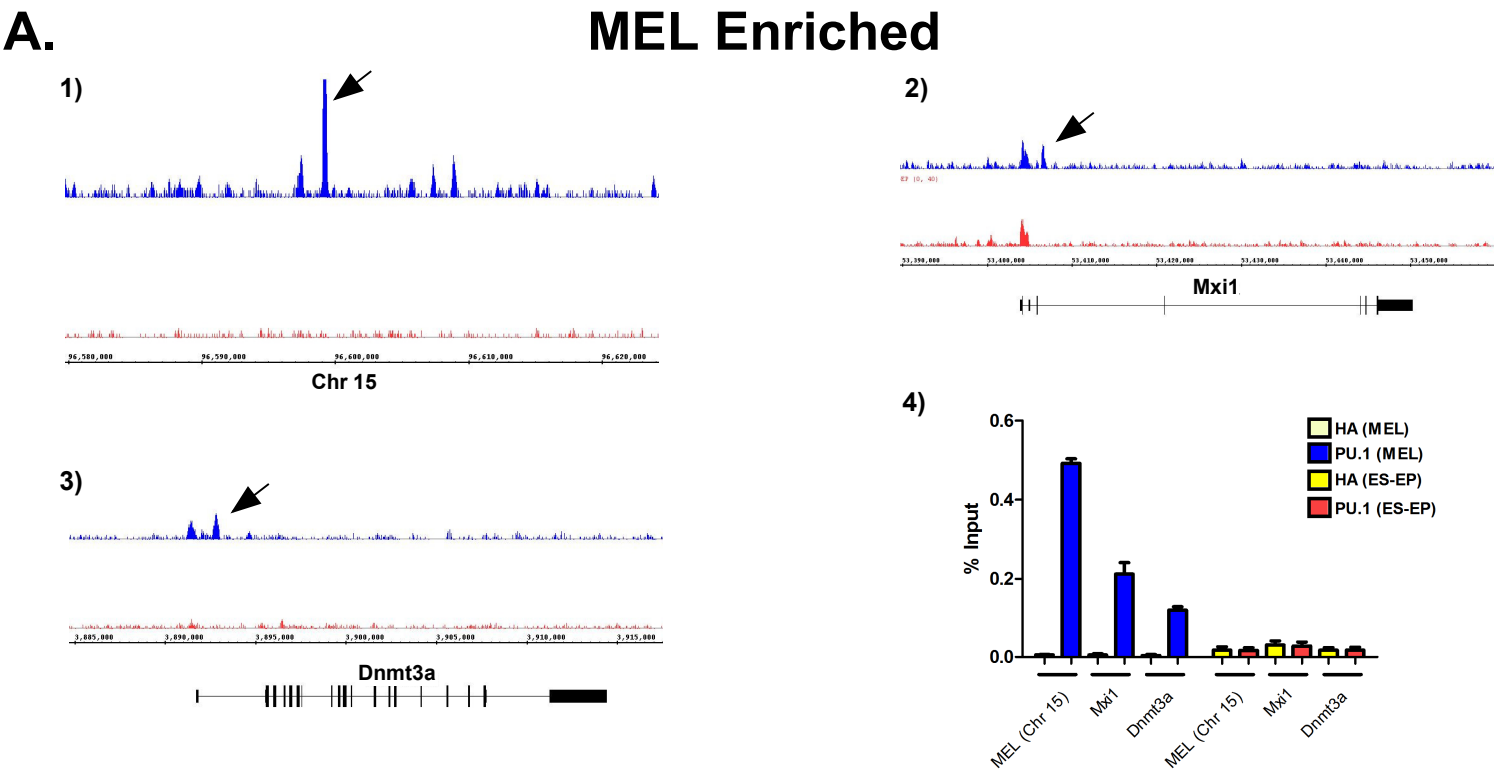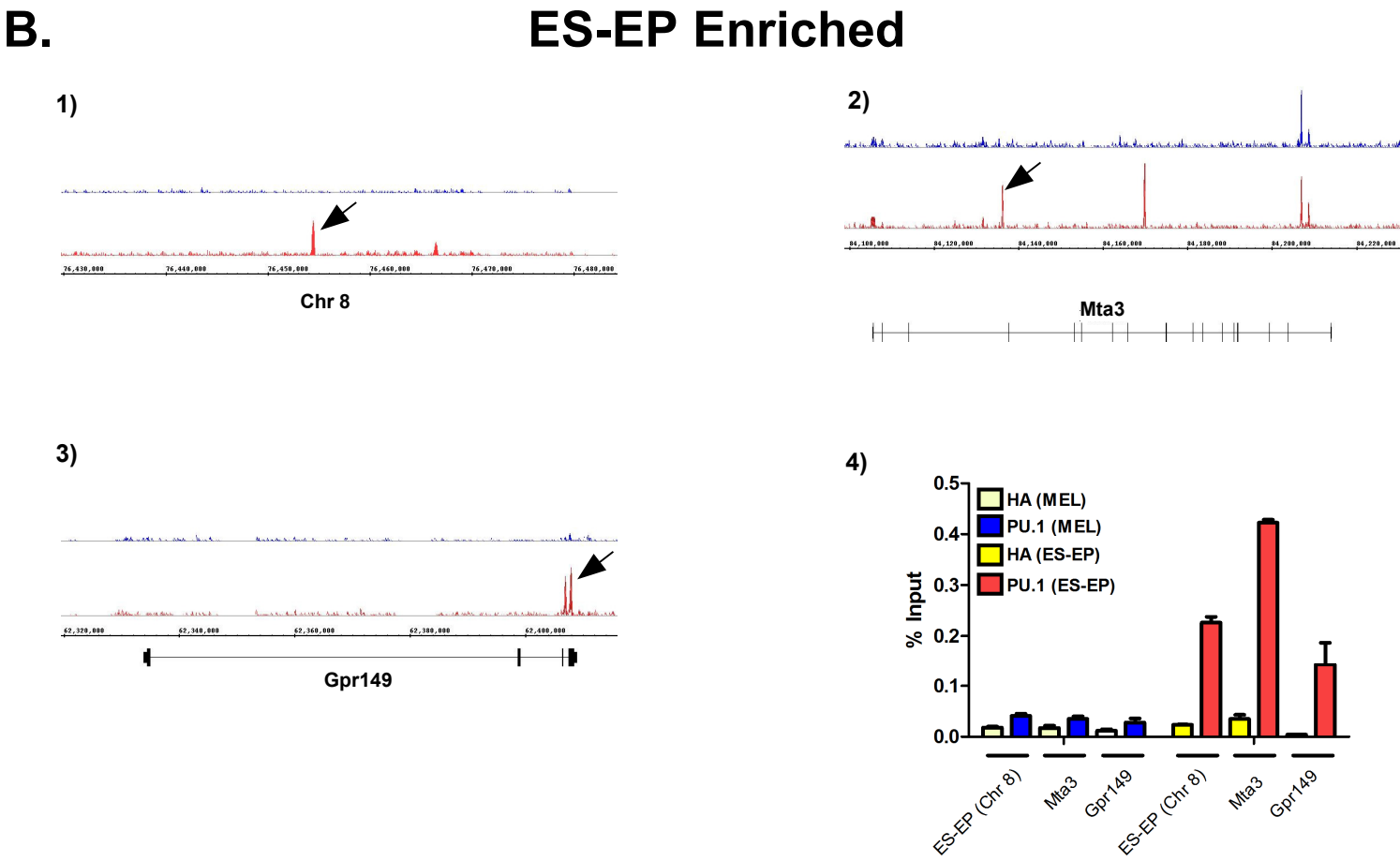

Supplement: Figure S1 — Comparisons of ChIP-Seq and qChIP data for loci differentially occupied by PU.1 in MEL cells and ES-EP. (A) Comparisons for loci exhibiting enriched occupancy of PU.1 in MEL cells. Panels 1–3 display signal tracks corresponding to 1) a gene-poor region on chromosome 15, 2) the region near the Mxi1 gene, 3) the region near the Dnmt3a gene. Panel 4 shows qChIP analyses near the peaks denoted with arrows in panels 1–3. A HA antibody was used as an isotype control. Sequences of qChIP primers are shown in Table S2. Standard deviations represent the errors from triplicate PCR reactions. Similar results were obtained with at least 2 independent chromatin preps. (B) As in (A) for loci exhibiting enriched occupancy of PU.1 in ES-EP. Panels 1–3 display signal tracks corresponding to 1) a gene-poor region on chromosome 8, 2) the region near the Mta3 gene, 3) the region near the Gpr149 gene. (0.41 MB PDF) [file pgen.1001392.s002.pdf]

Figure S2 (Wontakal et. al.)

A.

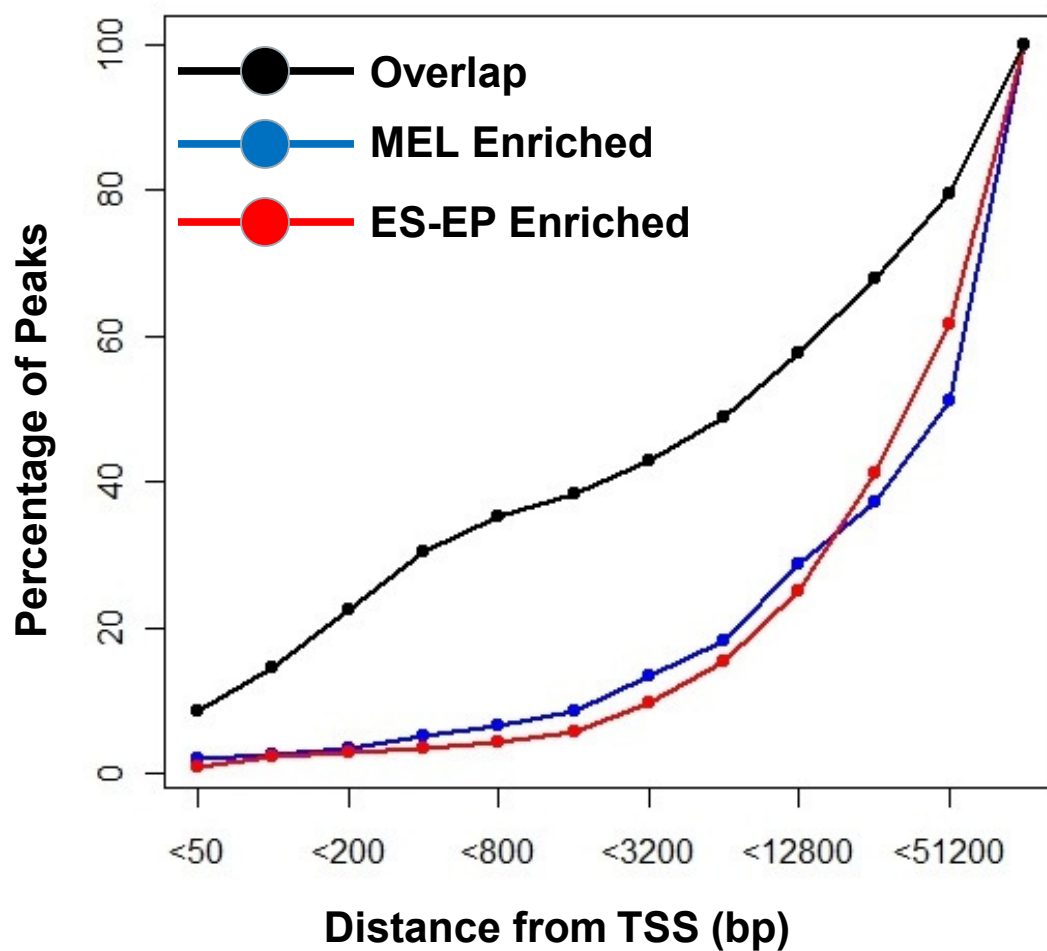

B.

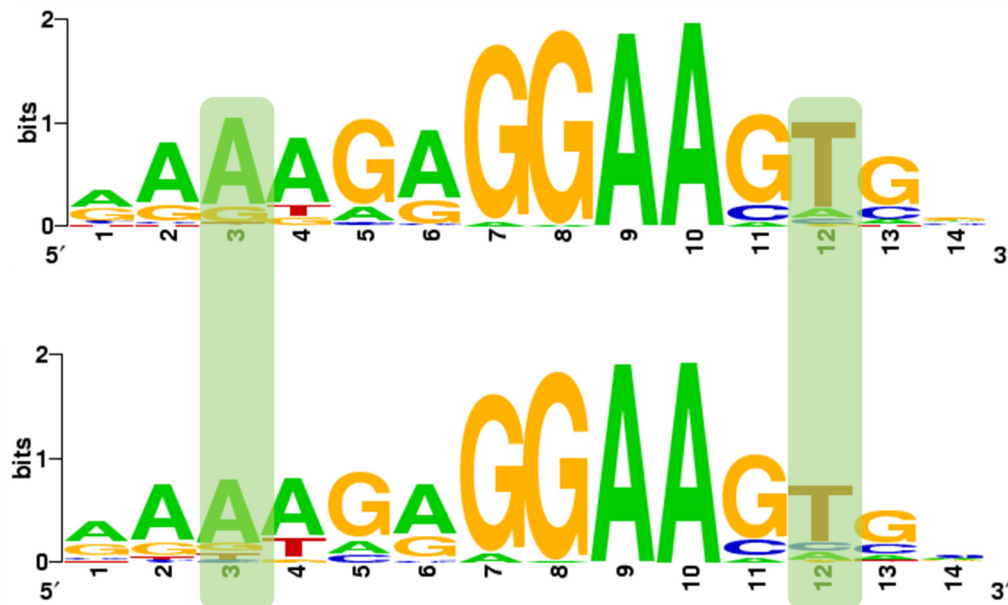

Supplement: Figure S2 — Properties of PU.1 ChIP-Seq peaks enriched in MEL cells or ES-EP. (A) The percentage of PU.1 ChIP-Seq peaks lying within the indicated distance from the closest TSS was calculated separately for peaks shared by the two cell types (Overlap) or enriched in either MEL cells or ES-EP, as described in Materials and Methods. (B) The derived position-weighted matrices from MEME analyses of PU.1 ChIP-Seq peaks enriched in MEL cells (top) and ES-EP (bottom) are shown. The thymidine [T] and adenosine [A] residues that differ relative to matrices derived from MEME analyses of all PU.1 ChIP-Seq peaks in each cell type (Figure 2B) are highlighted. (0.27 MB PDF) [file pgen.1001392.s003.pdf]

**Figure S3 (Wontakal et. al.)**

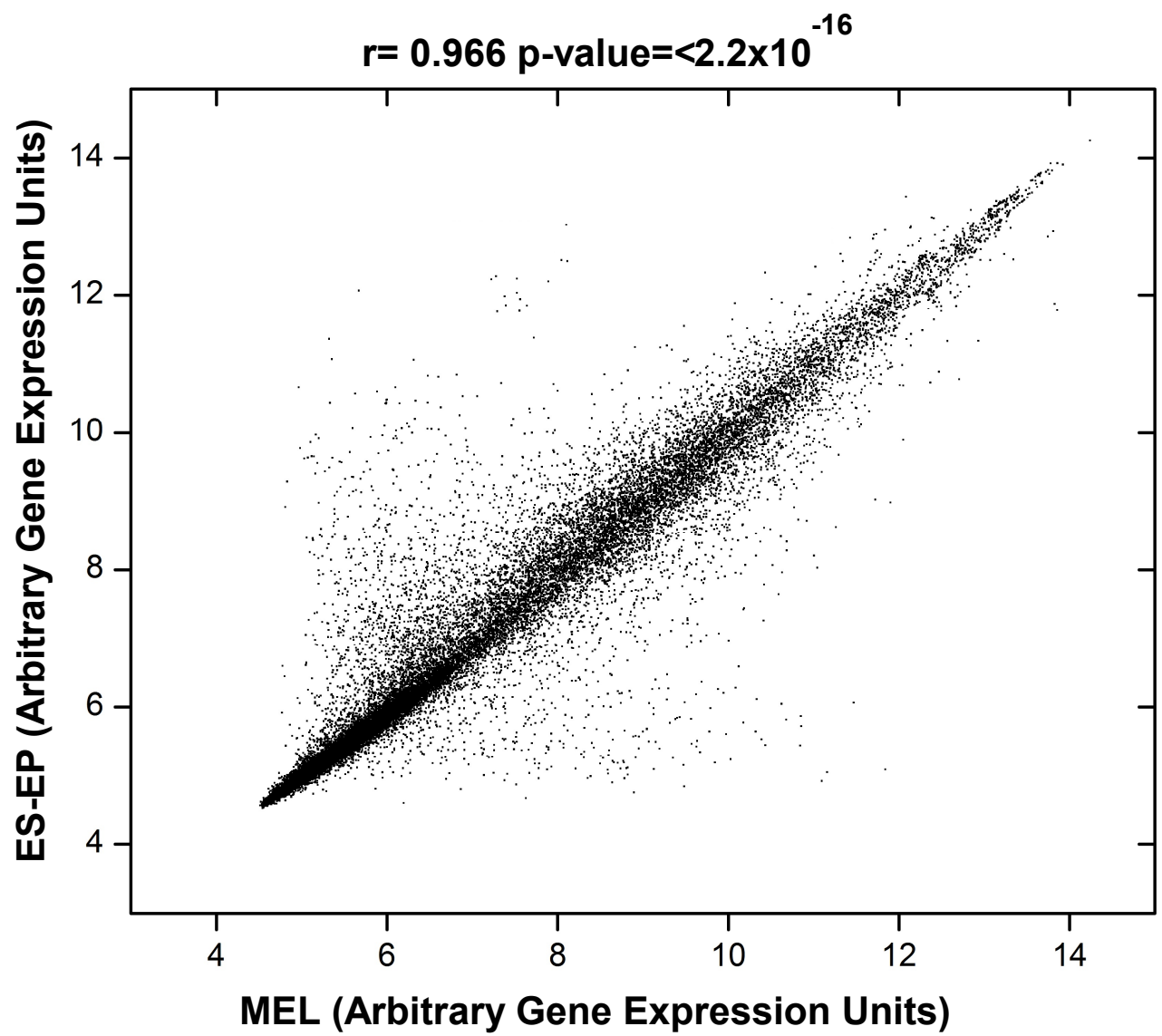

Supplement: Figure S3 — High correlation of gene expression in MEL cells and ES-EP. Gene expression levels were obtained separately for MEL cells and ES-EP with the Affymetrix microarray platform. After data processing and normalization, the relative expression of genes in the two samples are shown here as a scatter plot with larger units representing higher expression. The Pearson's correlation coefficient and its statistical significance are shown at the top of the figure. (0.77 MB PDF) [file pgen.1001392.s004.pdf]

Figure S4 (Wontakal et. al.)

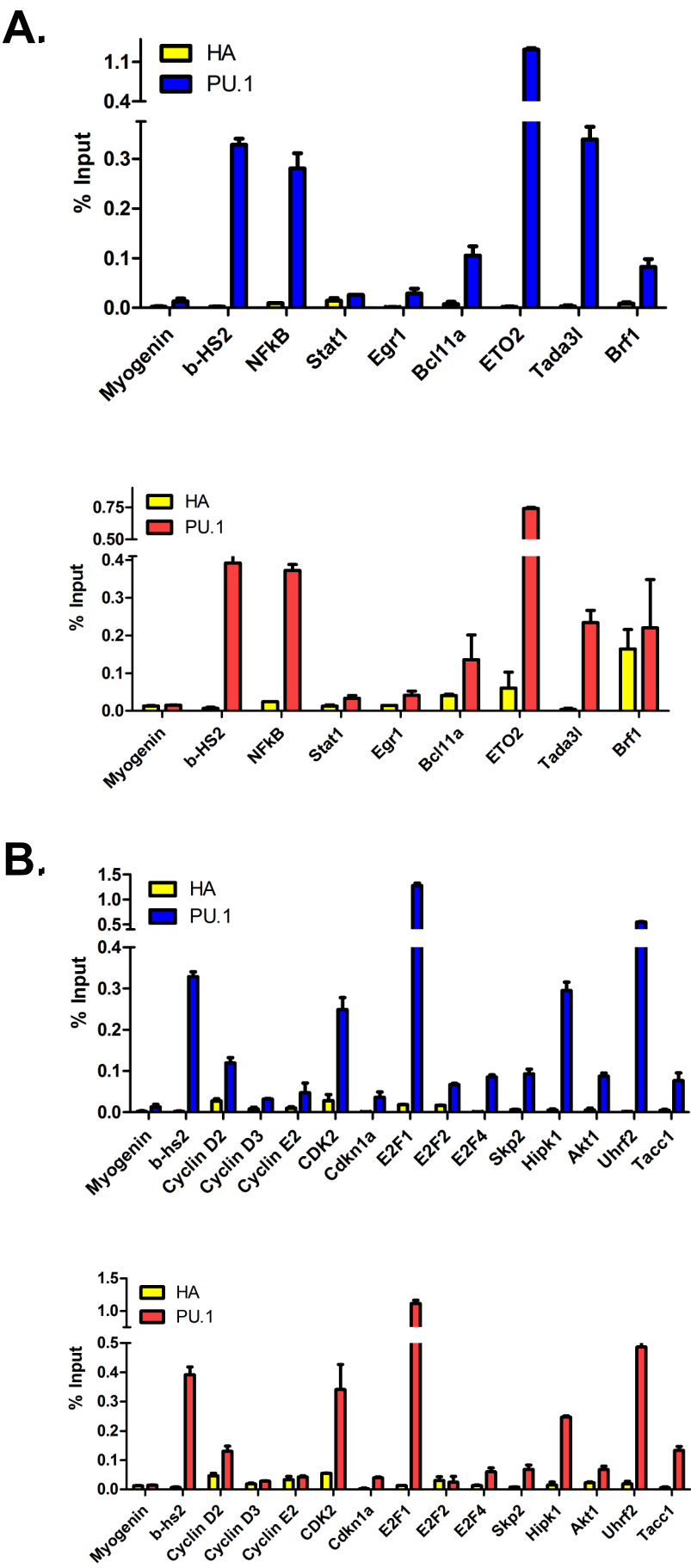

Supplement: Figure S4 — qChIP validation of PU.1 occupancy near genes involved in gene regulation and cell cycle regulation. (A) and (B) qChIP was performed as described in Materials and Methods with chromatin from MEL cells (top) and ES-EP (bottom) with primers described in Table S2. The genes analyzed represent examples of genes from the IPA gene expression (A) and cell cycle (B) categories that have PU.1 ChIP-Seq peaks within +/−2 kb of their TSS. Myogenin and β-HS2 serve as negative and positive controls, respectively. A HA antibody was used as an isotype control. Standard deviations were calculated from triplicate PCR reactions. Similar results were obtained with at least two independent chromatin preparations. (0.16 MB PDF) [file pgen.1001392.s005.pdf]

Figure S6 (Wontakal et. al.)

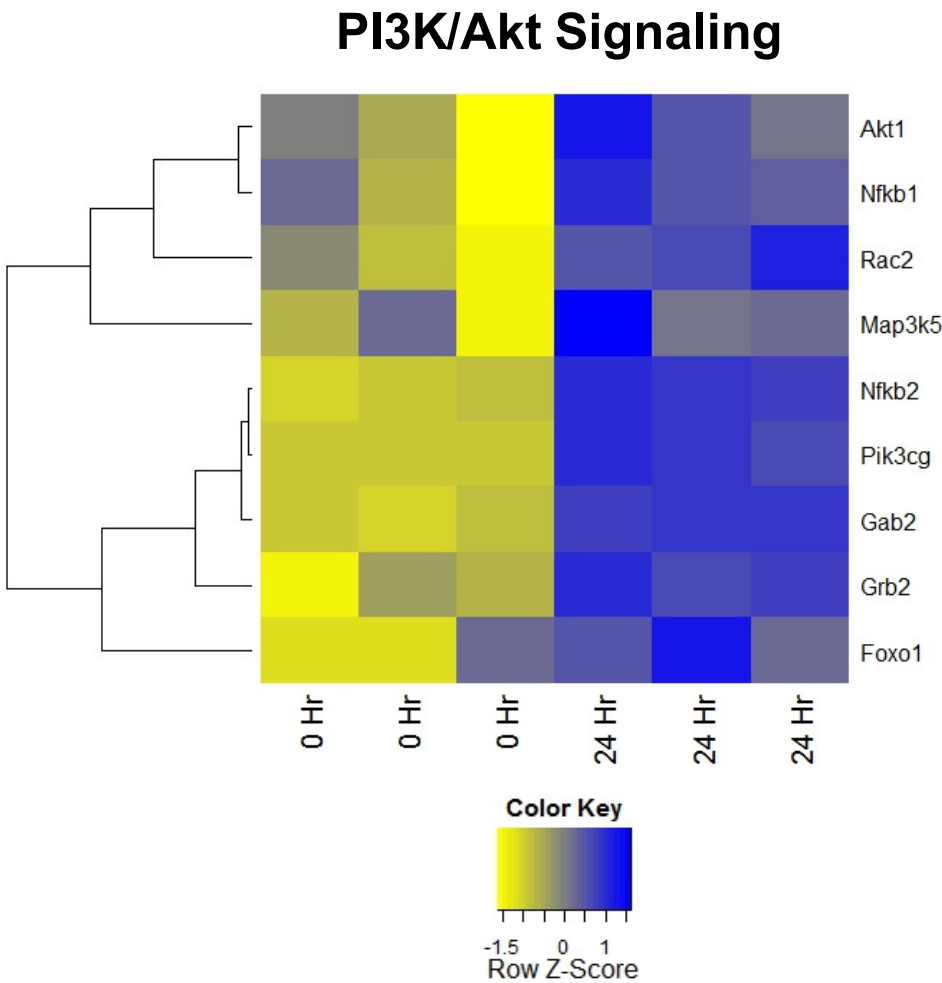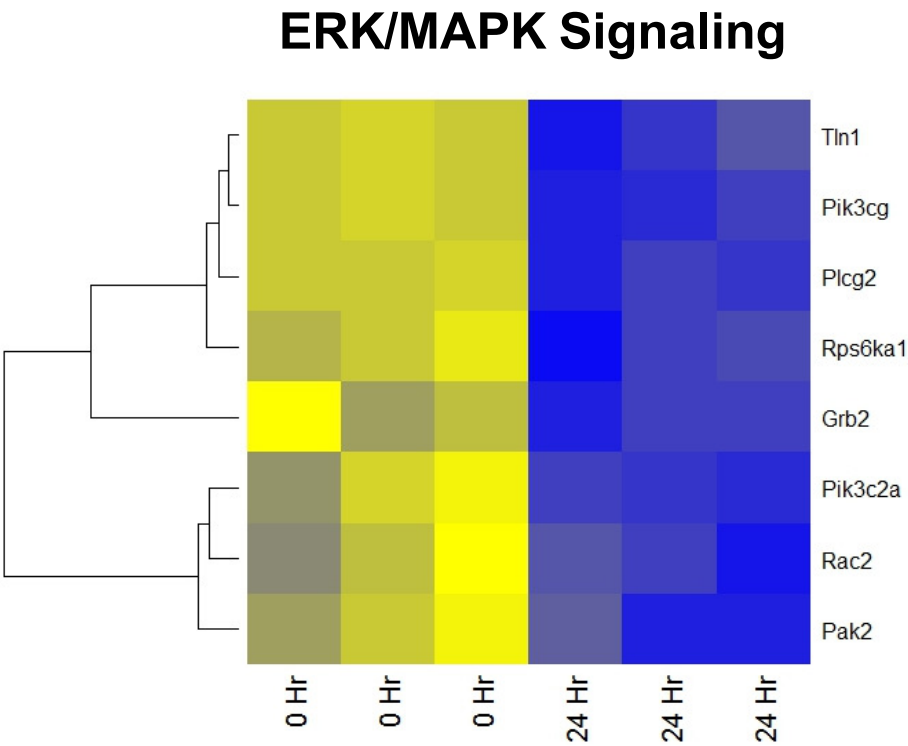

Supplement: Figure S6 — PU.1 promotes PI3K/Akt and ERK/MAPK signaling in macrophages. A previously published [49] gene expression analysis of PU.1−/− cells induced to differentiate into macrophages was used to generate heatmaps depicting the response of the indicated genes in the PI3K/Akt and ERK/MAPK signaling pathways to the activation of PU.1. The genes shown in this analysis are the same as the genes found to be regulated by PU.1 in MEL cells (Figure 5). (0.11 MB PDF) [file pgen.1001392.s007.pdf]
