# Supplementary material for: A Large Gene Network in Immature Erythroid Cells Is Controlled by the Myeloid and B Cell Transcriptional Regulator PU.1
Source: PLoS Genet. 2011 Jun 9;7(6):e1001392. doi: 10.1371/journal.pgen.1001392 (PMC3111485; doi:10.1371/journal.pgen.1001392)

# Figure S5 (Wontakal et. al.)

A.

**Total Genes**

| Motif | Observed | Expected | $Log_2$Ratio |
|---|---|---|---|
| ZF5 | 50% | 14% | 1.82 |
| E2F | 38% | 9.5% | 2.01 |
| AP2 | 30% | 5.6% | 2.42 |
| ETF | 22% | 0.49% | 5.51 |
| CHCH | 28% | 5.0% | 2.47 |
| GC | 22% | 3.4% | 2.68 |
| Sp1 | 22% | 3.6% | 2.58 |
| ABI4 | 19% | 3.1% | 2.59 |
| Movo-B | 16% | 2.0% | 3.03 |
| Muscle | 15% | 5.3% | 1.53 |

B.

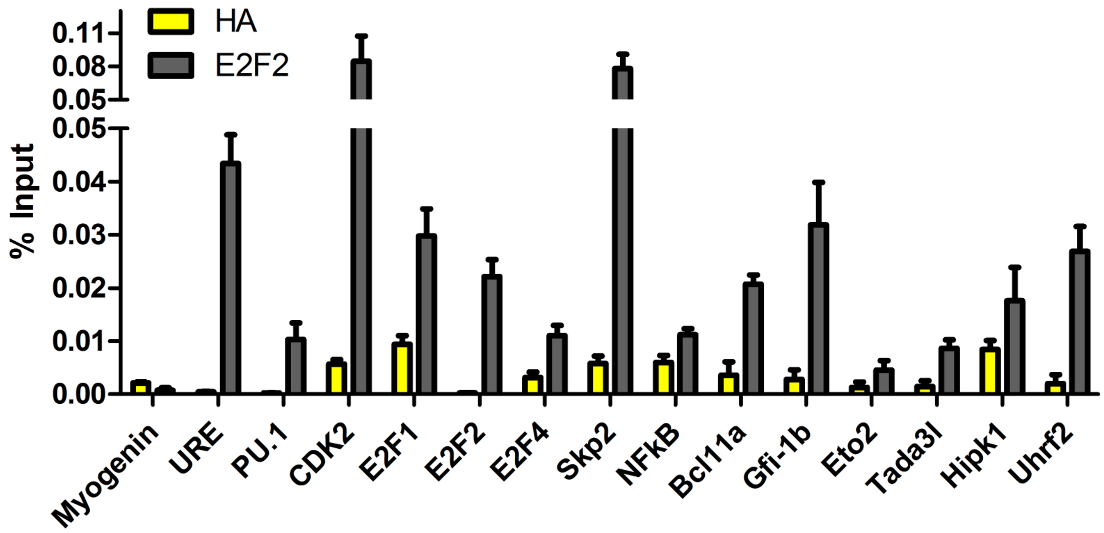


C.

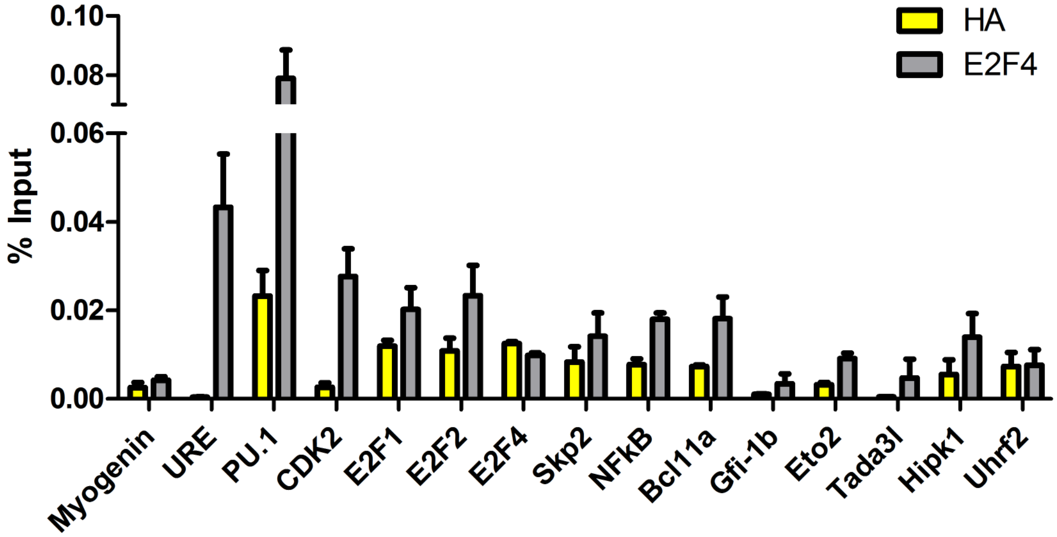

Supplement: Figure S5 — E2F factors occupy PU.1 target genes in MEL cells. (A) DNA sequences from PU.1 ChIP-Seq peaks within the proximal promoter in both ES-EP and MEL cells were analyzed using the TRANSFAC database as described in Materials and Methods. The names of the ten most significant observed transcription factor motifs (p-value<10−5) that were found in at least 10% of peaks and ≥2 fold enriched over the expected frequency are shown. The ratio between observed frequency and expected frequency is represented by a log2ratio. (B) and (C) qChIP analysis of E2F2 (B) and E2F4 (C) occupancy was performed as described in Materials and Methods at the indicated PU.1 target genes in MEL cells. A HA antibody was used as an isotype control. Standard deviations were calculated from triplicate PCR reactions. Similar results were obtained with at least two independent chromatin preparations. The myogenin locus serves as a negative control. (0.16 MB PDF) [file pgen.1001392.s006.pdf]
