# Supplementary material for: A Large Gene Network in Immature Erythroid Cells Is Controlled by the Myeloid and B Cell Transcriptional Regulator PU.1
Source: PLoS Genet. 2011 Jun 9;7(6):e1001392. doi: 10.1371/journal.pgen.1001392 (PMC3111485; doi:10.1371/journal.pgen.1001392)
Supplement: Table S1 — Results of Ingenuity Pathway Analysis of PU.1 Target Genes. Ingenuity Pathway Analysis was performed on genes with one or more PU.1 ChIP-Seq peaks within 2 kb of TSS in both MEL and ES-EP. The ten most significant molecular and cellular functions identified are listed in descending order of significance. The number of genes associated with a given function and bound by PU.1 is shown. p-values have been corrected for multiple testing using the Holm-Bonferroni method. (0.01 MB DOCX) [file pgen.1001392.s008.docx]

**Table S1. Results of Ingenuity Pathway Analysis of PU.1 Target Genes**

|  | **Molecular and Cellular Functions** | **Number of Genes Bound** | **p-Value** |
| --- | --- | --- | --- |
| **1.** | Gene expression | 1081 | 1.3x10^-27^ – 1.3x10^-1^ |
| **2.** | Cell cycle | 687 | 3.1x10^-24^ – 1.3x10^-1^ |
| **3.** | DNA replication, recombination, and repair | 440 | 1.5x10^-12^ – 1.3x10^-1^ |
| **4.** | RNA post-transcriptional modification | 176 | 5.0x10^-12^ – 1.3x10^-1^ |
| **5.** | Molecular transport | 167 | 5.1x10^-12^ – 1.3x10^-1^ |
| **6.** | Protein trafficking | 171 | 5.1x10^-12^ – 1.3x10^-1^ |
| **7.** | Cell death | 1197 | 1.8x10^-11^ – 1.3x10^-1^ |
| **8.** | Post-translational modification | 591 | 1.4x10^-9^ – 1.3x10^-1^ |
| **9.** | Cellular growth and proliferation | 783 | 1.7x10^-7^ – 1.3x10^-1^ |
| **10.** | Protein degradation | 145 | 6.4x10^-8^ – 1.3x10^-1^ |
