# Supplementary material for: A Large Gene Network in Immature Erythroid Cells Is Controlled by the Myeloid and B Cell Transcriptional Regulator PU.1
Source: PLoS Genet. 2011 Jun 9;7(6):e1001392. doi: 10.1371/journal.pgen.1001392 (PMC3111485; doi:10.1371/journal.pgen.1001392)
Supplement: Table S2 — Primers used in this study. (0.02 MB DOCX) [file pgen.1001392.s009.docx]

**Table S2. Primers used in this study**

| **Gene** | **Forward Primer** | **Reverse Primer** | **Reference** |
| --- | --- | --- | --- |
| **Myogenin** | GAA TCA CAT GTA ATC CAC TGG A | ACG CCA ACT GCT GGG TGC CA | [[1](#_ENREF_1)] |
| **β-HS2** | TGT GTT CAG CCT TGT GAG CCA GC | TGG ACT TCC TCC TAG AGA CCC AG | [[2](#_ENREF_2)] |
| **URE** | GGG AGG CAG AGC ACA CAT G | GTT TCC ACA TCG GCA GCA G | [[3](#_ENREF_3)] |
| **PU.1** | GTA GCG CAA GAG ATT TAT GCA AAC | GCA CAA GTT CCT GAT TTT ATC GAA | [[3](#_ENREF_3)] |
| **c-myb** | TCA TCC TTC TCC CGA GTG TC | AGA CCC CGA CAC AGG TAA TG | This study |
| **Fli-1** | CGG TCT GGC TGA CAA GTA AA | CGA GCG AGT TGG AGG AGT AG | This study |
| **Cdkn1a** | TCT GTG TAC GTG CGT GTG TG | TAA ATT CCC GCC TAT GTT GG | This study |
| **Klf1** | TCC ATG CAG TAG CCA GTG AG | TCC TCC TTG AGC AGA AGA GC | This study |
| **EpoR** | GTC CAA AGG ACC TAG CAC CA | GAT CGA GCT CCC AGA GAC TG | This study |
| **CDK2** | CAA CTT TCC CGC AGT TCC TGG TTT | GAG ACT CTC TTG TCC AGG TGG AAT | This study |
| **E2F1** | GCA AGC CAG CAG ACA TCA GTT CAA | CAG GCT TTG GCA CCA AAT TCC CAA | This study |
| **E2F2** | TAC ACT TCG CTT TAC ACG CAG ACG | ACC CAG ACA CGT ATA CGG GAA GTA | This study |
| **E2F4** | ACT GCC GGT ACT GCT CAC TAA CTT | ACA ACT CCT TAG GGC AGC TAG GAA | This study |
| **Skp2** | AAC TAG CAA CGT TCC ATC ACC ACC | AAT CAG AGT GGA AGA ACC CAG GCA | This study |
| **NFkB** | TTC TTA GGA GCT GGG AAG GGC TTT | TGC ACA GCC AGA TTT CAC CAA AGG | This study |
| **Bcl11a** | AAA CTT GGG CTG GAT TTC TCT CGC | TTG AGA GCT GAG CCT CCA AGT TGA | This study |
| **Gfi-1b** | ACC TTA AAG GGA AGC AGA CAC CCT | GCA GAG ACT CAT AAC GTT GAC CGA | This study |
| **Eto2** | GCT TTT CCA ATC AGC AGG TC | CTT CCC CAC AAA CAC CAA CT | This study |
| **Tada3l** | TAC AAC CAA AGC GAC AAG CA | GGA GGG AGA GCC GAG AGA TA | This study |
| **Hipk1** | TAG CGC CAT GAC ACC TAC TG | TAA ACG ACC TCG GAG ACT GC | This study |
| **Uhrf2** | GCC ATG GCT CCT TCC TAT TT | GCG AGG ACT GAC GCT CTT AG | This study |
| **Stat1** | TTA CGC AGG CAC TGC AAA TCC AAG | TGC ATT AAG TAG GCG CAT CAC TGC | This study |
| **Egr1** | CCT TCC ATA TTA GGG CTT CCT GCT | CGA ATC GGC CTC TAT TTC AAG GGT | This study |
| **Akt1** | CAC TGC GCA CAC CAA CAG | CTC GGA GTA GGA GCA GGA AG | This study |
| **Tacc1** | CTG CCG CTA GGA CTT AAT GG | CAA ATC CTG GGC GTA ACA AG | This study |
| **Brf1** | CGG GAC CGA TAA AGG AAC TC | CAG GTT TGG AGC TTT GCT TC | This study |
| **Cyclin D2** | TAA CTT TCA AGC TGT TGT CCG CCC | AGA AAG GTT TCT GCA GGA GGG TCA | This study |
| **Cyclin D3** | ACC AGC GTG TCC TGC AGA GTT TA | TTA CCT CCA GCA TCC AGT ATG CCA | This study |
| **Cyclin E2** | CGG GCC CGG CCT ATA TAT TGA GTT | TGG TTC CCT TAG CTC CCG CAT | This study |
| **MEL (Chr 15)** | TTC CTC TTT CTG AGT GGC AGG TGT | ATT GAG TCC GCA GGT CGC TGA TAA | This study |
| **Mxi1** | GGG AAG GAC CAT GCT GTA GAA GAA | TCT GAA CAC GCA GGT TCT GGG AAA | This study |
| **Dnmt3a** | TCC AGG GCT TAG AGA CCA AAT GCT | TAG AAT ACT CAG CCT GGC AGC ACA | This study |
| **ES-EP (Chr 8)** | TTC TGT ACT GAG TGT GTG CCA GGA | GCT TGC AGT TCC TCA TCC AGC AAA | This study |
| **Mta3** | TAG CAT TCC TCG ACC GTT TGC TCA | AAT GAG GAA GTC AGA ACC GGG CAT | This study |
| **Gpr149** | TTG GGT GGA AGG ATT CTG AG | CGT AGC AGG TGC AGA CAG AG | This study |
