# Supplementary material for: A Large Gene Network in Immature Erythroid Cells Is Controlled by the Myeloid and B Cell Transcriptional Regulator PU.1
Source: PLoS Genet. 2011 Jun 9;7(6):e1001392. doi: 10.1371/journal.pgen.1001392 (PMC3111485; doi:10.1371/journal.pgen.1001392)
Supplement: Text S1 — Supplemental Materials and Methods. (0.06 MB DOC) [file pgen.1001392.s010.doc]

**Supplemental Materials and Methods**

*Cell Culture and differentiation*

GATA-1/ER cells were cultured in DMEM supplemented with 10% Fetal bovine serum (Gemini), 2mM L-glutamine (Invitrogen), 100 units/ml penicillin, 100 μg/ml streptomycin, and 5μg/ml puromyocin. Cells were grown at 37^0^ in a humidified, 5% CO_2_ atmosphere. U2OS, HeLa and 293T cells were grown under the same conditions except that puromyocin was omitted and 10% CO_2_ was used. 32D cells were also cultured in similar conditions but DMEM was used with the addition of 10% WEHI IL-3 conditioned media and cells were maintained to less than 1x10^6^ cells/ml with daily media changes. ES-EP cells were cultured with StemPro34 media (Invitrogen) supplemented with 2u/ml Epogen (Amgen), 40ng/ml hIGF-1 (Sigma), 10^-6^M dexamethosone (Sigma), 100ng/ml murine SCF (R&D Systems/Invitrogen), and 0.1% β-mercaptoethanol (Gibco) at 37^o^ in a humidified 10% CO_2_ atmosphere. The cell concentration was maintained between 2x10^6^ and 6x10^6^ cells/ml by daily media changes.

*Analysis of early erythroid progenitor populations*

Mice with a deletion in an Upstream Regulatory Element (URE) that controls the expression of PU.1 were described previously . Fetal-livers from two URE^-/-^ mice, URE^+/-^ heterozygous, and wild-type (WT) animals were isolated between E13.5-15.5 as previously described . Erythroid populations were analyzed using flow cytometry with anti-mouse TER119-PE (eBioscience) and CD71-APC-Cy7 (eBioscience) antibodies following published protocols . The R1 population, CD71^med^ TER119^low^, was isolated using FACSAria II machine (BD Bioscience) and analyzed further.

*Antibodies and ChIP-Seq*

Antisera used in this study were: anti-PU.1 (Santa Cruz T21), anti-HA (Santa Cruz Y11), anti-E2F2 (Santa Cruz C-20), and anti-E2F4 (Santa Cruz C-108). Primers used in this study are described in Table S2.

For ChIP-Seq, duplicate, independent chromatin preparations were used. DNA was isolated from chromatin preparations prior to ChIP and used as an input DNA control. After immunoprecipitation, DNA was isolated and libraries were prepared for sequencing as described previously . Libraries were sequenced using Illumina Analyzer GAII and processed with the Illumina ELAND pipeline. Sequence reads were aligned to the mouse genome (mm9).

*ChIP-Seq Data Analysis*

Genome-wide binding profiles from replicate ChIP-Seq runs were highly correlated (data not shown), so we merged the data from duplicate sets for each cell type, and obtained a total of 12,710,420 and 13,416,531 uniquely mapped reads (up to two mismatches) from MEL and ES-EP cells, respectively. We also obtained 4,278,560 and 7,488,885 reads for input DNA controls for each respective cell type. We then applied two complementary software programs, cisGenome and spp , to identify PU.1 binding regions (i.e., peaks) by comparing reads in the immunoprecipitated (IP) sample to the input DNA sample. Peaks detected by either of these two programs at a false discovery rate (FDR) < 0.05 were merged. This resulted in the identification of a total of 17,668 peaks, 65.2% of which were detected by both programs. Of the 17,668 peaks, 14,032 were reported for MEL cells and 12,883 for ES-EP. However, further examination of the raw data of reads showed that most of the “cell-type specific” PU.1 peaks also contained ChIP-Seq signals from the alternative cell type. To account for this quantitative difference, and overcome the limitation introduced by thresholding in peak calling, we developed and applied an alternative strategy for detecting cell-type enriched PU.1 peaks more robustly. For each of the 17,668 peaks we counted the numbers of the reads from MEL and ES-EP samples, and then a peak was considered enriched in a given cell type if and only if it contained five times more reads (adjusted for read depths). Peaks with comparable numbers of reads (≤5 fold difference in reads) from the two samples were defined as shared. We chose fivefold based on our analysis of replicated ChIP-Seq runs (data not shown).

To investigate PU.1 global occupancy in the mouse genome, we calculated the frequencies of PU.1 peaks in the proximal promoter (± 2kb from TSS), distal end of genes (± 2kb from TES), within a gene body (from +2kb of TSS to -2kb of TES site), and otherwise intergenic regions, using Refseq gene annotations downloaded from the UCSC genome browser (mm9, July 2007) . PU.1 target genes were defined as genes with a PU.1 peak within the proximal promoter (defined as ± 2kb of its TSS).

For these PU.1 targets, Ingenuity Pathway Analysis (http://www.ingenuity.com/) was performed to identify enriched functional categories amongst these genes, as well as search for potential canonical pathways involving these genes. P-values were adjusted for multiple hypotheses testing using Benjamini-Hochberg correction. All statistical analyses were carried out in the R language.

*Microarray analysis*

The raw data (CEL files) from each microarray was normalized by RMA methods in GeneSpring GX software. The log2 transformed signal intensities were averaged for biological replicates, and the mean value was used to compute the fold expression change. Heat-maps were generated by calculating the mean value of all time points for a given gene and assigning a color gradient for each time point by calculating the log_2_ratio of that time point to the mean expression value. Gene expression data from PU.1^-/-^ cells differentiated into macrophages and expression data from PU.1 knockdown HSCs were downloaded from NCBI using accession numbers GSE131235 and GDS2411, respectively.

**Supplemental References**

1. Lu J, McKinsey TA, Zhang CL, Olson EN (2000) Regulation of skeletal myogenesis by association of the MEF2 transcription factor with class II histone deacetylases. Mol Cell 6: 233-244.

2. Stopka T, Amanatullah DF, Papetti M, Skoultchi AI (2005) PU.1 inhibits the erythroid program by binding to GATA-1 on DNA and creating a repressive chromatin structure. EMBO J 24: 3712-3723.

3. Hoogenkamp M, Krysinska H, Ingram R, Huang G, Barlow R, et al. (2007) The Pu.1 locus is differentially regulated at the level of chromatin structure and noncoding transcription by alternate mechanisms at distinct developmental stages of hematopoiesis. Mol Cell Biol 27: 7425-7438.

4. Rosenbauer F, Wagner K, Kutok JL, Iwasaki H, Le Beau MM, et al. (2004) Acute myeloid leukemia induced by graded reduction of a lineage-specific transcription factor, PU.1. Nat Genet 36: 624-630.

5. Zhang J, Socolovsky M, Gross AW, Lodish HF (2003) Role of Ras signaling in erythroid differentiation of mouse fetal liver cells: functional analysis by a flow cytometry-based novel culture system. Blood 102: 3938-3946.

6. Robertson G, Hirst M, Bainbridge M, Bilenky M, Zhao Y, et al. (2007) Genome-wide profiles of STAT1 DNA association using chromatin immunoprecipitation and massively parallel sequencing. Nat Methods 4: 651-657.

7. Ji H, Jiang H, Ma W, Johnson DS, Myers RM, et al. (2008) An integrated software system for analyzing ChIP-chip and ChIP-seq data. Nat Biotechnol 26: 1293-1300.

8. Kharchenko PV, Tolstorukov MY, Park PJ (2008) Design and analysis of ChIP-seq experiments for DNA-binding proteins. Nat Biotechnol 26: 1351-1359.

9. Rhead B, Karolchik D, Kuhn RM, Hinrichs AS, Zweig AS, et al. (2010) The UCSC Genome Browser database: update 2010. Nucleic Acids Res 38: D613-619.

10. Weigelt K, Lichtinger M, Rehli M, Langmann T (2009) Transcriptomic profiling identifies a PU.1 regulatory network in macrophages. Biochem Biophys Res Commun 380: 308-312.

11. Steidl U, Rosenbauer F, Verhaak RG, Gu X, Ebralidze A, et al. (2006) Essential role of Jun family transcription factors in PU.1 knockdown-induced leukemic stem cells. Nat Genet 38: 1269-1277.
